# Regular Dietary Intake of Palmitate Causes Vascular and Valvular Calcification in a Rabbit Model

**DOI:** 10.3389/fcvm.2021.692184

**Published:** 2021-06-23

**Authors:** Nathalie Donis, Zheshen Jiang, Céline D'Emal, Raluca Dulgheru, Martin Giera, Niek Blomberg, Philippe Delvenne, Alain Nchimi, Patrizio Lancellotti, Cécile Oury

**Affiliations:** ^1^Laboratory of Cardiology, Department of Cardiology, GIGA Institute, CHU Sart Tilman, University of Liège, Liège, Belgium; ^2^Center for Proteomics and Metabolomics, Leiden University Medical Center, Leiden, Netherlands; ^3^Department of Pathology, Centre Hospitalier Universitaire (CHU) University Hospital, Liège University, Liège, Belgium; ^4^Laboratory of Experimental Pathology, Groupe Interdisciplinaire de Géno-protéomique Appliquée (GIGA) Institute, Liege University, Liège, Belgium; ^5^Gruppo Villa Maria Care and Research, Maria Cecilia Hospital, Cotignola, Italy; ^6^Anthea Hospital, Bari, Italy

**Keywords:** palmitate, nutrition, blood fatty acids, vascular calcification, aortic valve calcification

## Abstract

**Aims:** Palmitic acid (PA) and oleic acid (OA) are two main dietary fatty acids. Dietary intake of PA has been associated with cardiovascular disease risk, and the effect of OA remains uncertain. Our study aimed to assess the effect of a short-term intake of lard, as source of PA and OA, on aorta and aortic valve.

**Methods and Results:** Rabbits were fed with two lard-enriched diets, containing either elevated levels of PA or of both PA and OA as compared to chow diet. After 16 weeks of each diet, calcification was observed in the aortic intima and in the aortic valve. The extent of calcification did not differ between the two diets. In contrast, rabbits fed chow diet did not develop any calcification. In blood, PA enrichment resulted in decreased lymphocyte and monocyte counts and increased levels of hemoglobin and haematocrit. Levels of the calcification inhibitor fetuin-A were also diminished, whereas creatinine levels were raised. Of note, none of the diets changed cholesterol levels in LDL or HDL. Comprehensive quantitative lipidomics analysis identified diet-related changes in plasma lipids. Dietary PA enrichment led to a drop of polyunsaturated fatty acids (PUFA), in particular of linoleic acid in cholesteryl esters, triglycerides and diacylglycerols (DAG). Ratios of PA to 18-carbon PUFA in DAG were positively correlated with the extent of aortic valve calcification, and inversely with monocyte counts. PA content in blood correlated with aorta calcification.

**Conclusions:** Regular dietary PA intake induces vascular and valvular calcification independently of traditional risk factors. Our findings raise awareness about PA-rich food consumption and its potential deleterious effect on cardiovascular health.

## Introduction

It is well-established that the risk of cardiovascular disease (CVD) is influenced by nutrition habits (1, 2). Dietary fatty acids, comprising saturated fatty acids (SFAs), monounsaturated fatty acids (MUFAs) and polyunsaturated fatty acids (PUFAs) are known to differentially affect CV health. It has been long considered that SFAs could increase the risk of CVD through their ability to raise low density lipoprotein cholesterol (LDL-C), whereas PUFAs could play a protective role by lowering total and LDL-C (3). Recently, it has been found that dietary SFAs do not necessarily increase the risk of CVD, which could be explained by various effects on LDL profiles (e.g., small dense LDL, large buoyant LDL, very low density lipoproteins, LDL oxidation) with distinct atherogenic effects (4), smaller size LDL particles being more likely to penetrate in vessel walls and contributing to atherosclerosis. It has now become clear that the food source and type of SFA are key in determining the risk of CVD (5, 6). Data from the Rotterdam Study revealed that a high risk of coronary artery disease (CAD) was associated with intake of palmitic acid (16:0) (PA), but not of SFA with other chain lengths (7). PA is the major SFA in palm oil, palm kernel oil and lard, the most widely consumed vegetable oils and animal fat, respectively. PA could be responsible for harmful cardiovascular effects by acting as a pro-inflammatory stimulus (8–11) and/or through direct effect on cardiovascular structures. An *in vitro* study indicates that PA promotes vascular calcification through direct effect on vascular smooth muscle cells (12, 13). The health impact of MUFAs is a matter of controversy (14). Oleic acid (18:1) (OA) is the most abundant MUFA, with a high content in olive oil. In the Multi-Ethnic study of Atherosclerosis, high plasma levels of OA correlated with coronary artery calcification, carotid plaque and aortic valve calcification. Most importantly, elevated OA levels were associated with CV events and all-cause mortality independently of typical risk factors (15). However, dietary OA hardly correlates with plasma levels of OA (16), and the impact of dietary OA on CV risk remains uncertain. PA and OA are two major daily food components. Yet, to date, there are no experimental studies that investigated whether regular PA or OA intake causes vascular and/or valvular calcification.

Our study aimed to assess the effect of a regular intake of a PA- and OA-rich food on rabbit aorta and aortic valve structures. Plasma lipids were then analyzed by comprehensive quantitative lipid analysis in order to identify diet-related changes in lipid profiles.

## Materials and Methods

### Ethic Statement and Animal Model

All rabbit experiments conform to the European Union guidelines for the care and use of laboratory animals and were conducted with protocols approved by the Animal Ethical Committee of the University of Liège (protocol number 1951). Twenty eight-week-old male New Zealand White rabbits (body weight 1.85 ± 0.17 kg) were purchased from Charles River (Charles River, France). Animals were kept in a temperature- and humidity-controlled environment in an air-conditioned room with standard rabbit chow and tap water *ad libitum*. After 2 weeks of acclimation, rabbits were randomly assigned in three different groups. They received the following diets for 16 weeks. The control group was composed of seven rabbits that received standard rabbit chow. The 0.175% group (0.175%) was composed of seven rabbits that received standard rabbit chow supplemented with 1.75 g of lard/kg. The 5% group (5%) was composed of six rabbits that received standard rabbit chow supplemented with 50 g of lard/kg. Lard-supplemented chows were produced by Safe company (Safe, France). Lard contained 23.8% PA and 41.2% OA. Body weight was measured on a weekly basis. Blood draws and echocardiography were performed at baseline, mid-protocol and end-protocol. Aortic valve and left ventricular functions were assessed by echocardiography using a neonatal 12S probe and a VividTM E95 system (GE Healthcare). Anesthesia was induced prior to any procedure (except body weight measurements) by intramuscular injections of droperidol (0.625 mg/kg), xylazine (5 mg/kg) and ketamine (35 mg/kg) in hind legs. Euthanasia was conducted in anesthetized animals by intracardiac injection of pentobarbital (200 mg/kg).

### Histological Analyses of Rabbit Heart

Hearts were harvested, weighed and fixed in 4% paraformaldehyde–PBS solution for 24 h before dehydration into 70% ethanol solution overnight at room temperature. One heart from a rabbit of the chow diet group was lost during the procedure. They were then embedded in paraffin wax and sectioned at 7 μm. Tissue structure was studied on hematoxylin-eosin stained sections. Tissue calcification was assessed on alizarin red stained sections (Supplementary Figure 1). Quantification of aorta and aortic valve calcified areas was conducted on alizarin stained heart sections using QuPath, an open-source software designed for histopathology slide analysis (17). We used the random forest method. This algorithm is a robust supervised machine learning method for pixel classification. Classification was based on calcified and non-calcified areas of available sections from rabbit aorta showing macrocalcifications that were detectable by computed tomography.

### Blood Processing

At each blood draw, differential white blood cell counts, red blood cell indices, platelet count and mean platelet volume were measured in 3.8% citrated-anticoagulated whole blood on a Cell-Dyn 3700 hematocytometer (Abbott Laboratories). EDTA and 3.8% citrated plasma aliquots were prepared through two successive centrifugation steps at 1,700 g at room temperature and stored at −80°C. Serum aliquots were prepared via a 30-min rest to allow blood clotting followed by one centrifugation step at 1,700 g at room temperature. They were stored at −80°C until further analysis.

### Biomarker Measurements

Measurements of fetuin-A and IL-6 were performed in serum by enzyme-linked immunosorbent assays according to the manufacturer's recommendations (fetuin-A: MyBiosource, IL-6: Cusabio). Inactive dp-ucMGP level was measured in EDTA plasma through an automated chemiluminescence sandwich immunoassay on the IDS-iSYS Multi-Discipline Automated System (ImmunoDiagnosticSystems). Measurements of calcium, phosphorus, iron, transferrin, creatinine, total cholesterol, triglycerides, LDL-c, HDL-c, ApoA and ApoB were performed in serum by the use of the AU480 Chemistry Analyzer (Beckman Coulter).

### Lipidomics

Lipidomics analysis was carried out on the commercial Lipidyzer^TM^ platform, according to the manufacturer's instructions with some modifications (Sciex) (18, 19). The Lipidyzer platform is a targeted, quantitative lipidomics platforms, monitoring more than 1.000 lipid species from 13 lipid classes (CE, cholesterol ester; CER, ceramides; DAG, diacylglycerides; DCER, dihydroceramides; FFA, free fatty acids; HCER, hexosylceramides; LPC, lysophosphatidylcholine; LPE, lysophosphatidylethanolamine; PC, phosphatidylcholine; PE, phosphatidylethanolamine; SM, sphingomyelin; and TAG, triacyltriglycerides). The platform is a flow-injection based approach based on differential mobility separation of lipid classes with subsequent MRM scanning of individual lipid species on a QTrap 5500 mass spectrometer equipped with SelexION technology (Sciex). Quantification is based on isotope dilution, incorporating 54 isotopically labeled standards. Each lipid species is corrected by the closest deuterated IS within its lipid class, in terms of carbon and double bond number of the fatty acid side chain. Subsequently, the obtained area ratio is multiplied by the concentration of the respective IS and corrected for volume and weight; as a consequence, this quantitation can be considered accurate within a specified quantitative bias. For a detailed technical description of the method and its quantitative nature, please refer to Alarcon-Barrera et al. (19), Contrepois et al. (20), and Cao et al. (21). Briefly, lipid analysis was performed in 3.8% citrated plasma after methyl-tert-butyl ether (MTBE) extraction in flow-injection mode, separating lipid classes by differential mobility spectroscopy (22) followed by tandem mass spectrometry of lipid species with a QTrap 5500 operated in multiple reaction monitoring mode. Lipid species were identified and quantified on the basis of characteristic mass spectrometric transitions. Commercial Lipidyzer software automatically calculated lipid species concentrations. All samples were analyzed in a randomized fashion. Control plasma samples, blanks and pooled study samples were assessed daily as quality controls. To 25 μL of rabbit plasma was added 25 μL of Lipidyzer internal standard mix. Six hundred microliters MTBE and 150 μL methanol was added and the samples were vortex and stored at room temperature for 30 min. Subsequently, samples were centrifuged at 16.100 × g for 5 min. Seven hundred and fifty microliters of the organic extract was transferred to a fresh 2 mL Eppendorf tube. Lipid extraction was repeated adding 300 μL MTBE and 100 μL methanol to the original sample. Samples were vortexed and centrifuged for 15 min at 16.100 × g. Three hundred and fifty microliters was transferred and organic extracts combined. For phase separation, 300 μL of water was added to the combined organic extracts and samples were spun for 5 min. at 16.100 × g. Seven hundred microliters of the organic extract was subsequently transferred to a glass vial and dried under a gentle stream of nitrogen. The dried organic extract was re-dissolved in 250 μL Lipidyzer running buffer [10 mM ammonium acetate in (50:50 [v/v]) methanol:dichloromethane] and transferred to a glass micro-vial insert. Samples were analyzed on the Lipidyzer platform.

### Statistical Analysis

Readouts of the Lipidyzer™ platform were lipid class concentration (nmol/g), lipid species concentration (nmol/g), specific fatty acid concentration by lipid class (nmol/g) and the corresponding compositional data (%) of all previously mentioned items. The entire lipidomic datasets are shown in Supplementary Table 1. Missing data were imputed with 10% of the lowest data measured. Between-diet comparison was performed by ANOVA and *post-hoc* Dunnett's test with log2 transformed data. Normality of variables was tested by Shapiro-Wilk test. Over time changes in blood cells and circulating biomarkers were assessed by either paired *t*-test or Wilcoxon signed-rank test when appropriate. Correlations between calcification, lipid data and circulating biomarkers were analyzed by either Pearson or Spearman method and results were shown with corresponding correlation coefficient (Pearson r or Spearman ρ). Data are presented as median (interquartile range [p25–p75]). All tests were performed 2-sided and *P* < 0.05 was considered significant. All statistical analyses were performed using SAS 9.4 (SAS Institute, Cary NC).

## Results

### Regular Intake of PA Causes Mild Calcification of Aortic Valve and Aorta

Rabbits were fed with a standard chow diet or diets enriched with 0.175% and 5% lard diet groups. Diet compositions are shown in Table 1. Proteins and nitrogen free extracts did not differ between the three diets. The 0.175% lard diet contained PA levels similar to average consumption by children aged 3 to 14 years (CCAF 2013 survey, France). In this diet, PA accounted for most SFA. It contained twice less OA compared to chow diet. PA/PUFA, SFA/PUFA and MUFA/PUFA ratios were increased by about 2-fold, while the ratio of OA/PUFA was slightly lower than in chow diet. The 5% lard diet contained higher levels of both PA and OA, which resulted in elevated ratios of PA (7.4-fold) and OA (3-fold) to PUFA as compared to chow diet.

**Table 1:** Composition of chow, 0.175% and 5% lard diets in terms of energy (%Kcal) and fatty acid content.

	**Chow**	**0.175% lard**	**5% lard**
Energy (% Kcal)			
Proteins	22.69	21.20	18.00
Lipids	10.14	7.60	21.60
Nitrogen free extracts	67.17	71.20	60.40
Fatty acids (mg/kg)			
PA	3,100	5,417	25,494
Total SFA	5,500	5,985	29,108
OA	6,500	3,216	20,600
Total MUFA	7,600	9,792	31,830
Total PUFA	10,100	6,448	11,006
Fatty acid ratios			
SFA:PUFA	0.54	0.93	2.64
MUFA:PUFA	0.75	1.52	2.89
PA:PUFA	0.31	0.84	2.32
OA:PUFA	0.64	0.50	1.87

*MUFA, monounsaturated fatty acid; OA, oleic acid; PA, palmitic acid; PUFA, polyunsaturated fatty acid; SFA, saturated fatty acid*.

**Figure 1:**
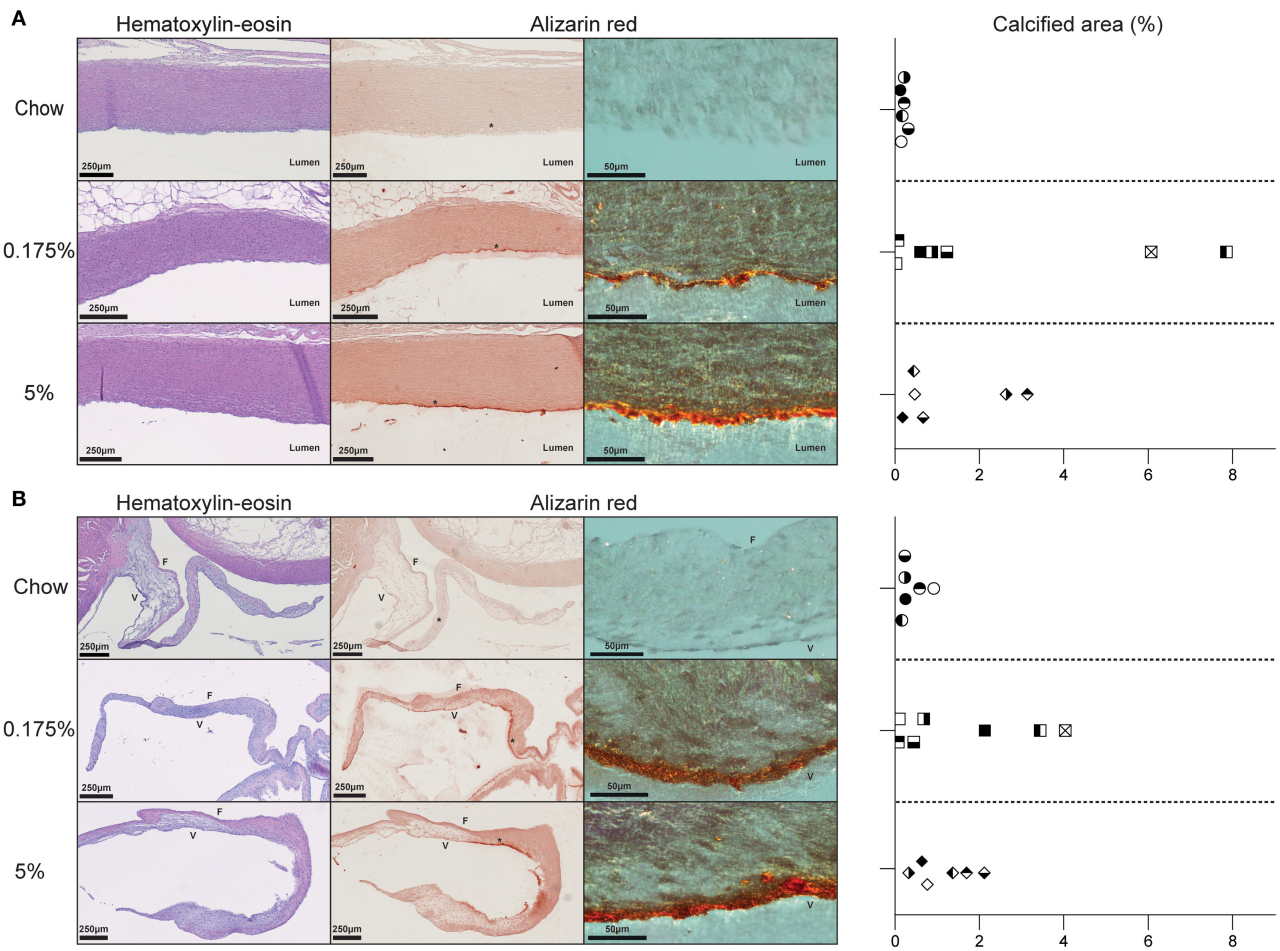
Effect of PA and PA/OA enriched diets on rabbit aorta and aortic valve structures. **(A)**. Representative pictures of aortic wall for chow, 0.175% and 5% lard diet groups stained with hematoxylin-eosin or alizarin red. Graph represents the percentages of calcified areas in aorta in all three rabbit groups. **(B)**. Representative pictures of aortic valve of chow, 0.175% and 5% lard diet groups stained with hematoxylin-eosin, hematoxylin-eosin or alizarin red. Graph represents the percentages of calcified areas in aortic valve in all three rabbit groups. Alizarin red pictures are taken with light microscopy (left panels) and with polarized light microscopy at higher magnification of ^*^ zone (right panels). In graphs, one symbol corresponds to one rabbit.

After 16 weeks of diets, body and heart weights remained unchanged between groups. Echocardiographic examination did not reveal any vascular or valvular functional alteration. We then performed histological analyses of heart sections. Hematoxylin-eosin staining of heart sections from rabbits fed with lard-containing diets did not show any vascular or valvular structural modification as compared to control rabbits (Figures 1A,B). However, alizarin red staining of serial sections revealed calcium deposits in arterial and valvular tissues in both 0.175% and 5% lard groups (Figures 1A,B and Supplementary Figure 1). Whole tissue of large arteries and aortic valve appeared reddish by optical microscopy. We observed microcalcifications in the aortic media as well as calcification lining the aortic intima and the ventricularis side of the aortic valve. There were no calcium deposits in the control group. Computational quantification of alizarin red-stained calcium deposits indicated variable percentages of calcified area in aorta and aortic valves among rabbits fed with lard diets, which did not differ between the 0.175% and the 5% lard diet groups (Figures 1A,B). Thus, these results indicate that a slight elevation of dietary PA triggers a calcification process in aorta and aortic valve. Higher levels of both PA and OA in diets does not increase this process further.

### Influence of PA- and PA/OA-Enriched Diets on Blood Cells and Circulating Biomarkers

We investigated whether the 0.175% and 5% lard diet groups provoked changes in blood cells, inflammation, calcification biomarkers, and minerals after 16 weeks (Table 2). The 0.175% lard diet caused a decrease of lymphocyte, monocyte and basophil counts, which led to an increase of platelet-to-lymphocyte ratio, of neutrophil percentage and neutrophil-to-lymphocyte ratio. In the 5% lard group, the monocyte counts similarly decreased and the neutrophil percentage increased, but the lymphocyte counts were not modified. Levels of hemoglobin and haematocrit raised in the two lard diet groups together with a decrease of the calcification inhibitor fetuin-A and phosphorous, and an increase of creatinine levels. In contrast, we did not observe any changes in dephosphorylated-uncarboxylated Matrix Gla-protein (dp-ucMGP), nor significant induction of the inflammatory cytokine, interleukin-6 (IL-6). Hence, blood biomarkers are consistent with the observed mild calcification induced by the two diets. Modifications in circulating blood cells induced by the 0.175% lard diet are compatible with the occurrence of a low-grade inflammatory response.

**Table 2:** Levels of circulating biomarkers measured before (pre) and after (post) 16 weeks of PA or PA/OA-enriched diets.

	**0.175%**			**5%**		
	**Pre**	**Post**	***P-*value**	**Pre**	**Post**	***P-*value**
White blood cell count (K/μl)	4.98 (3.86–5.16)	2.32 (1.24–3.16)	**<0.001**	4.81 (2.39–5.03)	2.80 (1.62–3.24)	0.069
Neutrophil count (K/μl)	0.79 (0.52–0.94)	0.93 (0.76–1.17)	0.087	0.88 (0.78–1.05)	0.98 (0.82–1.07)	0.478
Neutrophil percentage (%)	13.70 (11.80–22.00)	70.00 (23.80–71.90)	**0.006**	21.00 (15.90–55.60)	33.60 (26.30–63.30)	**0.006**
Lymphocyte count (K/μl)	3.70 (2.85–4.02)	0.63 (0.24–2.18)	**<0.001**	3.42 (0.54–3.61)	1.74 (0.58–2.23)	0.088
Lymphocyte percentage (%)	77.60 (71.70–79.10)	27.00 (22.10–69.00)	**0.007**	68.95 (39.60–76.60)	62.15 (35.90–69.40)	0.317
Monocyte count (K/μl)	0.24 (0.19–0.32)	0.07 (0.04–0.09)	**0.001**	0.24 (0.04–0.37)	0.04 (0.02–0.07)	**0.042**
Monocyte percentage (%)	5.81 (4.61–6.33)	2.89 (1.46–5.47)	**0.021**	6.25 (2.52–8.33)	1.33 (0.72–3.45)	0.095
Eosinophil count (K/μl)	0.001 (0.000–0.002)	0.001 (0.000–0.001)	0.500	0.000 (0.000–0.000)	0.002 (0.000–0.003)	0.054
Eosinophil percentage (%)	0.02 (0.00–0.05)	0.03 (0.00–0.05)	0.651	0.00 (0.00–0.00)	0.05 (0.00–0.09)	0.052
Basophil count (K/μl)	0.11 (0.09–0.16)	0.00 (0.00–0.11)	**0.018**	0.21 (0.01–0.28)	0.06 (0.01–0.08)	0.094
Basophil percentage (%)	2.66 (2.06–3.46)	0.10 (0.00–3.57)	0.212	4.19 (0.27–5.52)	1.66 (0.49–2.28)	0.219
Red blood cell count (M/μl)	5.57 (5.36–5.92)	5.98 (5.89–6.10)	0.078	5.70 (5.43–6.09)	5.87 (5.46–6.08)	0.171
Hemoglobin (g/dl)	11.00 (10.10–11.50)	12.40 (11.40–12.70)	**<0.001**	11.45 (10.70–11.70)	11.95 (11.30–12.30)	**<0.001**
Hematocrit (%)	35.50 (32.90–38.00)	39.80 (37.00–40.50)	**0.016**	37.70 (35.10–38.40)	39.05 (37.20–39.90)	**<0.001**
Platelet count (K/μl)	312.00 (278.00–325.00)	306.00 (281.00–328.00)	0.172	315.50 (280.00–384.00)	294.00 (278.00–313.00)	0.197
Mean platelet volume (fl)	3.65 (3.28–3.90)	3.77 (3.57–3.88)	0.583	4.08 (3.33–4.56)	3.86 (3.43–4.03)	0.356
Platelet-to-lymphocyte ratio	87.84 (75.87–108.73)	488.04 (165.32–938.27)	**0.032**	105.82 (90.30–500.00)	179.32 (140.36–461.41)	0.319
Neutrophil-to-lymphocyte ratio	0.17 (0.15–0.33)	2.60 (0.34–3.24)	**0.021**	0.29 (0.23–1.46)	0.54 (0.37–1.77)	0.399
dp-ucMGP (pmol/l)	436.63 (403.40–595.22)	350.16 (322.90–453.57)	0.062	351.30 (328.68–438.14)	379.41 (300.00–475.18)	0.539
IL-6 (pg/ml)	505.99 (247.82–985.14)	39.66 (27.46–101.45)	0.229	60.95 (24.24–83.85)	43.93 (15.28–105.42)	0.789
Fetuin-a (ng/ml)	70.85 (62.37–79.73)	55.15 (46.92–63.05)	**0.004**	63.92 (51.05–65.56)	51.16 (40.31–56.47)	**0.001**
Creatinine (mg/dl)	0.57 (0.52–0.58)	0.72 (0.61–0.81)	**0.003**	0.58 (0.55–0.67)	0.79 (0.69–0.84)	**0.007**
Transferrin (g/l)	1.12 (1.04–1.18)	1.05 (1.02–1.18)	0.501	1.13 (1.07–1.20)	1.12 (1.09–1.20)	0.63
Iron (μg/dl)	201.44 (183.66–206.30)	180.01 (172.21–223.87)	0.737	215.71 (184.95–222.70)	192.69 (180.74–237.25)	0.749
Calcium (mmol/l)	2.76 (2.72–2.82)	2.77 (2.49–3.00)	0.880	2.75 (2.64–2.87)	2.73 (2.61–2.82)	0.458
Phosphorous (mmol/l)	1.99 (1.81–2.07)	1.22 (0.97–1.25)	**<0.001**	2.26 (2.05–2.53)	1.27 (0.91–1.34)	**0.007**

*Values represent median (P25–P75). P-values < 0.05 are highlighted in bold*.

*dp-ucMGP, dephosphorylated uncarboxylated Matrix Gla Protein; IL-6, interleukin-6; NLR, neutrophil-to-lymphocyte ratio; PLR, platelet-to-lymphocyte ratio*.

### PA- and PA/OA-Enriched Diets Modify Plasma Levels of PA, OA, SFA, MUFA, and PUFA

**Figure 2:**
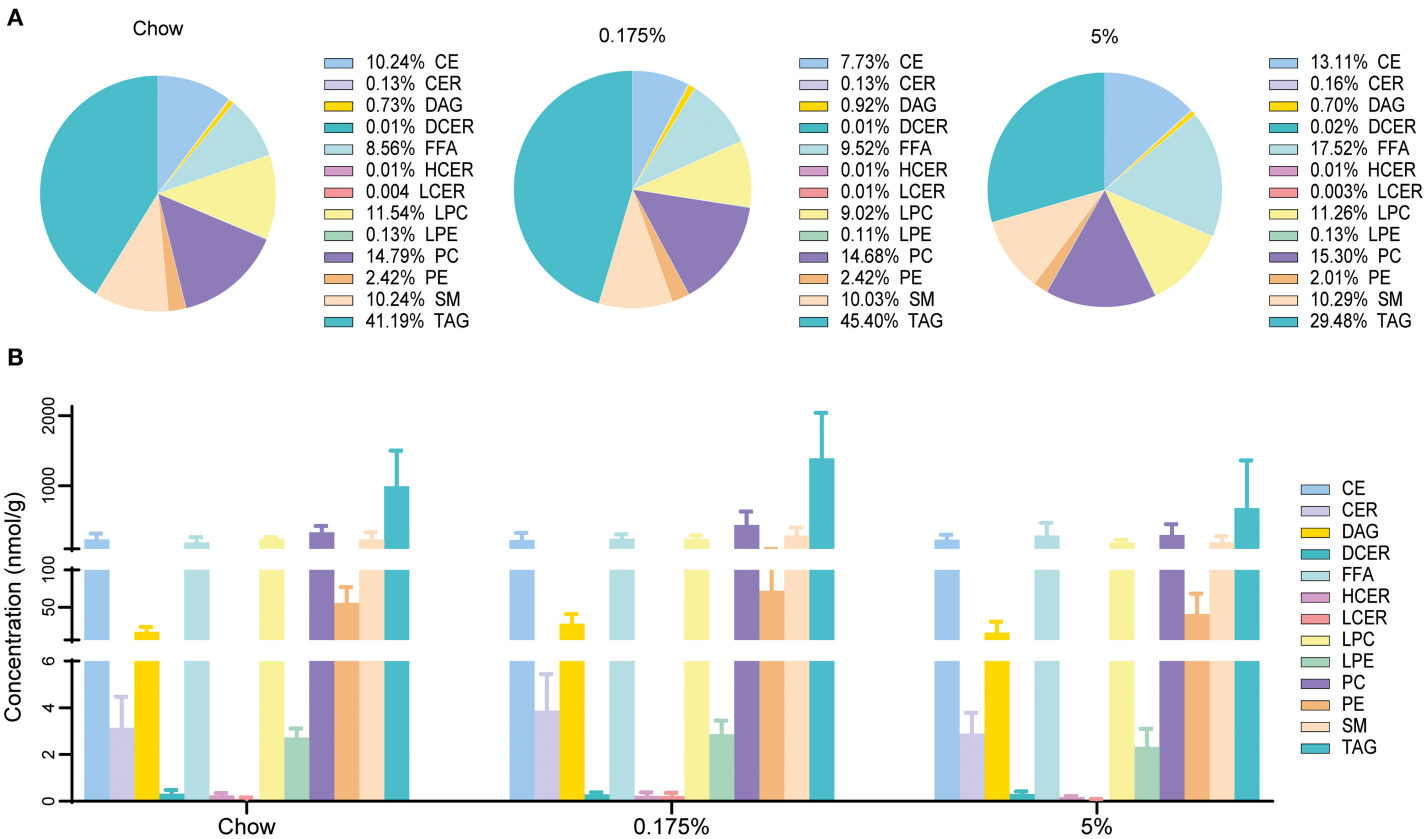
Lipid classes in plasma from rabbits fed chow, 0.175% and 5% lard diet groups. **(A)**. Proportion of lipid classes. **(B)**. Lipid class concentrations. CE, cholesteryl esters; CER, ceramides; DAG, diacylglycerols; DCER, dihydroceramides; FFA, free fatty acids; HCER, hesoxylceramides; LCER, lactosylceramides; LPC, lysophosphatidylcholines; LPE, lysophosphatidylethanolamines; PC, phosphatidylcholines; PE, phosphatidylethanolamines; SM, sphingomyelins; TAG, triacylglycerols.

**Figure 3:**
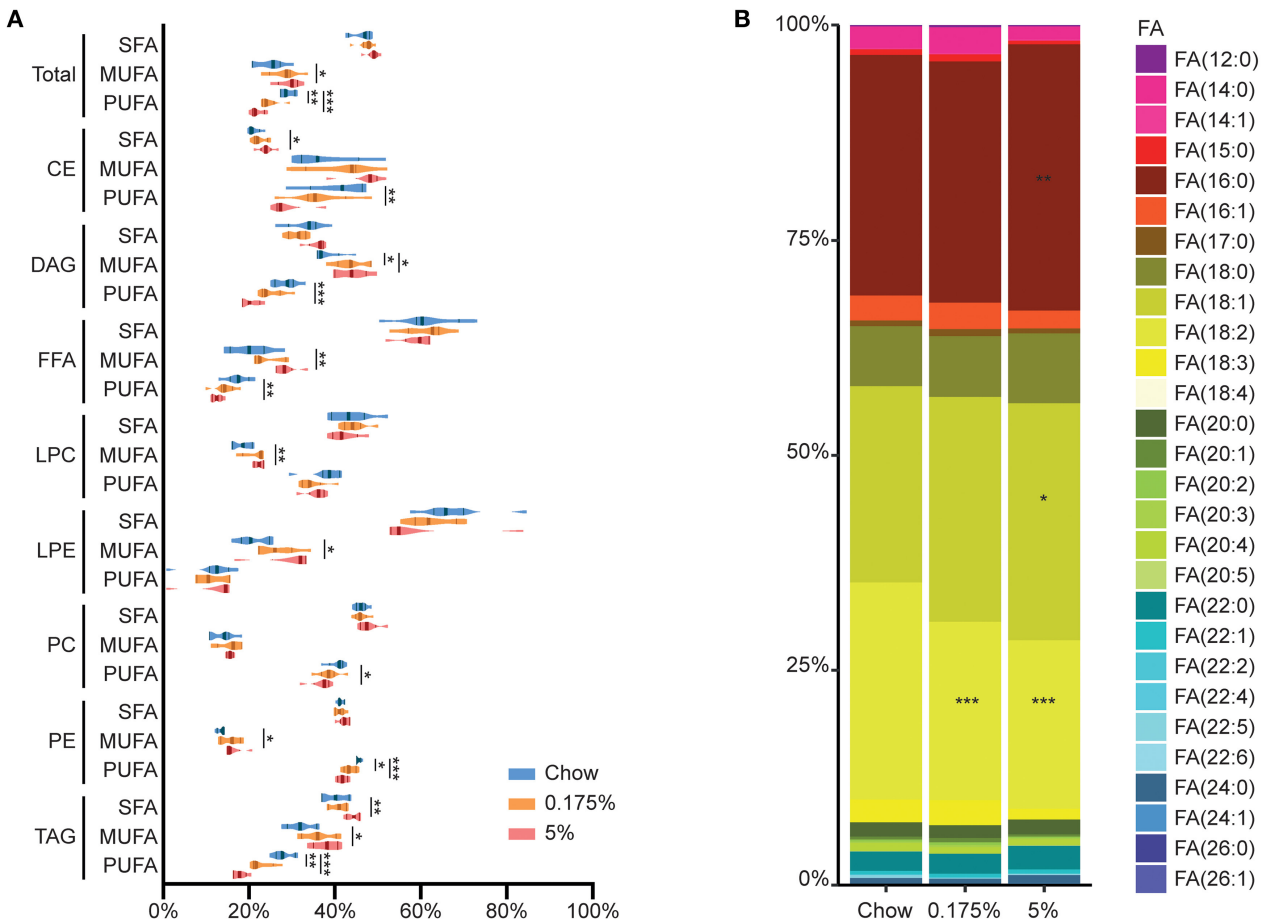
Detailed plasma lipid composition in chow, 0.175% and 5% lard diet groups. **(A)**. Percentages of SFA, MUFA and PUFA in total lipids and within lipid classes. Violin plot shapes represent value distribution within a group, with median shown by thick line and P25-P75 shown by thin lines. **(B)**. Representation of fatty acid percentages in total lipids. *P ≤ 0.05; **P ≤ 0.01; ***P ≤ 0.001 vs. chow.

Serum levels of conventional lipid parameters, e.g., total cholesterol, LDL, HDL, non-HDL, ApoB, ApoA-I and triglycerides, were not modified after 16 weeks of lard diets as compared to chow diet (Table 3). In order to assess the impact of the PA- and PA/OA-enriched diets on blood lipids and identify lipid profiles that could be associated with the observed vascular and valvular calcifications, we then performed a detailed quantitative lipidomics analysis on rabbit plasma samples. In total, 719 lipid species in 13 lipid classes were detected in the three rabbit groups (Supplementary Table 1). After 16 weeks of 0.175% and 5% lard diet groups, total levels of lipids within the different classes remained mainly unchanged as compared to control rabbits (Figure 2 and Supplementary Table 2). We only observed a decrease of lysophosphatidylcholine (LPC) concentration in the 5% lard diet as compared to chow diet. Nevertheless, we found striking changes in percentages MUFA and PUFA in total lipids and within several lipid classes (Figure 3A and Supplementary Table 2). The 5% lard diet resulted in elevation of total MUFA, whereas a drop in total PUFA percentages occurred in both 0.175% and 5% lard diet groups (23.93 and 21.44 vs. 28.55%, *P* = 0.002, *P* < 0.001). Within lipid classes, PUFA content decreased in cholesteryl esters (CE), diacylglycerols (DAG), triglycerides (TAG), in phospholipids, phosphatidylcholine (PC) and phosphatidylethanolamine (PE), and in free fatty acids (FFA) for the 5% lard group, and in PE and TAG for the 0.175% lard group (Supplementary Table 2). An increase of SFA content was observed in CE and TAG in the 5% lard group, whereas DAG, TAG, FFA, LPC, PE, LPE showed elevated content of MUFA. The 0.175% lard diet caused a rise of MUFA content in DAG.

**Table 3:** Effect of 0.175% lard and 5% lard diets on conventional lipid parameters.

	**Chow**	**0.175%**	***P-*value vs. chow**	**5%**	***P-*value vs. chow**
Total cholesterol (mg/dl)	24.08 (17.43–31.38)	23.09 (16.57–32.42)	0.962	21.24 (9.04–27.99)	0.703
Triglycerides (mg/dl)	92.49 (62.38–166.83)	166.60 (78.47–204.70)	0.630	42.55 (28.06–145.70)	0.255
LDL-c (mg/dl)	14.28 (8.83–18.59)	13.99 (9.45–18.65)	0.907	12.14 (5.79–18.97)	0.863
HDL-c (mg/dl)	5.95 (4.99–7.60)	5.14 (3.76–7.98)	0.894	5.85 (4.54–7.42)	1.000
Non HDL-c (mg/dl)	17.78 (11.00–25.92)	17.91 (10.01–27.56)	0.955	13.71 (5.32–20.57)	0.521
ApoA (mg/dl)	21.48 (20.20–22.33)	21.08 (19.70–22.43)	0.784	20.22 (19.47–20.72)	0.432
ApoB (mg/dl)	10.66 (10.36–11.38)	10.88 (10.44–11.99)	0.702	10.54 (9.62–10.84)	0.646

*Values represent median (P25–P75)*.

*HDL-C, high density lipoprotein cholesterol; LDL-C, low density lipoprotein cholesterol*.

Regarding the proportions of total fatty acids, we found increased content of total PA and OA in the 5% lard group as compared to chow diet (PA: 30.3 vs. 28.9%, *P* = 0.005; OA: 27.1 vs. 23.4%, *P* = 0.018). We also observed decreased linoleic acid content [FA (18:2)] both in the 0.175% and 5% lard groups compared to chow diet (19.6 and 18.9 vs. 24.8%, *P* < 0.001) (Figure 3B). When studied separately in each class, linoleic acid contents were decreased in CE, DAG and TAG in 5% lard group, and in TAG in the 0.175% lard group (Supplementary Table 2, Supplementary Figure 2).

Next, we calculated the plasma ratios SFA/PUFA, PA/OA, PA/PUFA and OA/PUFA in all three diet groups (Table 4). In total lipids, we observed increased SFA/PUFA ratios in the 0.175% lard group and in the 5% lard group as compared to the chow diet group. PA/PUFA and OA/PUFA ratios were also increased in both 0.175% lard and 5% lard groups. Changes in PA/PUFA ratios were reflected in TAG for the two groups, and in CE, DAG, and FFA for the 5% group. OA/PUFA ratios were increased in DAG, FFA, LPC, PE and TAG for the two groups, and in CE and PC for the 5% group. Of interest, in DAG, we found greatly increased PA/PUFA ratio in the 5% lard group that more particularly concerned PUFA with 18C-long FA chain [1.39 (1.35–1.52) vs. 0.86 (0.78–0.89), *P* < 0.001]. The ratio of SFA/PUFA with 18C-long FA chain was higher in the 5% lard diet than in chow diet [0.21 (0.17–0.21) vs. 0.12 (0.11–0.14), *P* < 0.001]. In TAG, a similar increase was observed for SFA/PUFA with 18C-long FA chain [0.24 (0.23–0.26) vs. 0.14 (0.13–0.16), *P* < 0.001] and PA/PUFA with 18C-long FA chain [2.13 (2.10–2.23) vs. 1.10 (1.06–1.33), *P* < 0.001].

**Table 4:** Effect of 0.175% lard and 5% lard diets on PA to OA, SFA to PUFA, PA to PUFA and OA to PUFA ratios in total lipids and lipid classes.

	**Chow**	**0.175%**	***P-*value vs. chow**	**5%**	***P-*value vs. chow**
PA:OA	1.20 (1.07–1.49)	1.10 (0.96–1.21)	0.148	1.12 (1.09–1.23)	0.429
SFA:PUFA	1.49 (1.39–1.52)	1.77 (1.71–1.89)	<0.001	2.16 (2.02–2.27)	<0.001
PA:PUFA	0.93 (0.91–1.01)	1.14 (1.02–1.18)	0.012	1.44 (1.25–1.51)	<0.001
OA:PUFA	0.79 (0.62–0.84)	1.11 (0.84–1.17)	0.014	1.25 (1.14–1.45)	<0.001
CE_PA:OA	0.43 (0.29–0.52)	0.35 (0.31–0.50)	0.914	0.36 (0.35–0.42)	0.780
CE_SFA:PUFA	0.53 (0.47–0.59)	0.61 (0.57–0.67)	0.200	0.83 (0.78–0.89)	<0.001
CE_PA:PUFA	0.34 (0.33–0.35)	0.38 (0.34–0.42)	0.312	0.56 (0.50–0.60)	<0.001
CE_OA:PUFA	0.79 (0.60–1.19)	1.11 (0.69–1.16)	0.640	1.65 (1.52–1.76)	0.016
DAG_PA:OA	0.71 (0.50–0.76)	0.57 (0.46–0.63)	0.124	0.69 (0.64–0.79)	0.827
DAG_SFA:PUFA	1.13 (1.10–1.31)	1.19 (1.07–1.44)	0.670	1.72 (1.71–1.81)	<0.001
DAG_PA:PUFA	0.85 (0.75–0.86)	0.78 (0.72–0.99)	0.741	1.34 (1.29–1.41)	<0.001
DAG_OA:PUFA	1.13 (1.10–1.32)	1.58 (1.36–1.82)	0.032	2.07 (1.68–2.29)	<0.001
FFA_PA:OA	2.56 (1.80–3.77)	2.17 (1.50–2.35)	0.222	1.59 (1.46–1.72)	0.018
FFA_SFA:PUFA	3.61 (3.01–4.43)	3.99 (3.85–4.76)	0.328	4.84 (4.35–5.38)	0.120
FFA_PA:PUFA	2.02 (1.67–2.56)	2.31 (2.25–2.66)	0.196	2.97 (2.66–3.31)	0.012
FFA_OA:PUFA	0.84 (0.68–1.06)	1.26 (1.14–1.55)	0.001	1.87 (1.82–1.93)	<0.001
LPC_PA:OA	1.72 (1.29–1.92)	1.31 (1.25–1.62)	0.274	1.24 (1.14–1.43)	0.037
LPC_SFA:PUFA	1.15 (0.99–1.26)	1.33 (1.11–1.39)	0.483	1.14 (1.08–1.26)	0.976
LPC_PA:PUFA	0.70 (0.66–0.83)	0.84 (0.74–0.88)	0.401	0.74 (0.70–0.82)	0.878
LPC_OA:PUFA	0.48 (0.39–0.51)	0.59 (0.56–0.66)	0.021	0.60 (0.57–0.62)	0.009
LPE_PA:OA	1.05 (0.85–1.35)	0.92 (0.73–0.98)	0.236	0.64 (0.59–0.83)	0.076
LPE_SFA:PUFA	5.47 (4.23–6.35)	6.00 (3.92–7.58)	0.791	3.79 (3.55–4.53)	0.801
LPE_PA:PUFA	1.89 (1.26–2.21)	2.07 (1.40–2.35)	0.852	1.46 (1.30–1.56)	0.886
LPE_OA:PUFA	1.80 (1.40–2.10)	2.18 (1.89–2.68)	0.986	2.19 (2.02–2.69)	0.822
PC_PA:OA	2.47 (2.14–2.91)	1.99 (1.88–2.60)	0.188	2.19 (2.04–2.34)	0.218
PC_SFA:PUFA	1.13 (1.09–1.19)	1.21 (1.10–1.27)	0.503	1.26 (1.18–1.34)	0.031
PC_PA:PUFA	0.82 (0.71–0.90)	0.80 (0.73–1.01)	0.826	0.90 (0.80–1.02)	0.291
PC_OA:PUFA	0.33 (0.26–0.38)	0.39 (0.33–0.46)	0.142	0.42 (0.39–0.42)	0.043
PE_PA:OA	0.65 (0.62–0.74)	0.60 (0.51–0.76)	0.393	0.60 (0.56–0.64)	0.202
PE_SFA:PUFA	0.91 (0.88–0.92)	0.95 (0.92–0.98)	0.054	1.02 (0.98–1.04)	<0.001
PE_PA:PUFA	0.20 (0.18–0.20)	0.23 (0.19–0.24)	0.240	0.23 (0.21–0.25)	0.056
PE_OA:PUFA	0.30 (0.27–0.30)	0.36 (0.29–0.42)	0.042	0.36 (0.35–0.41)	0.004
TAG_PA:OA	1.08 (0.99–1.34)	1.02 (0.81–1.09)	0.150	1.04 (0.99–1.18)	0.831
TAG_SFA:PUFA	1.43 (1.31–1.56)	1.76 (1.65–2.01)	0.003	2.49 (2.37–2.62)	<0.001
TAG_PA:PUFA	1.05 (1.03–1.22)	1.29 (1.20–1.48)	0.014	2.03 (1.96–2.15)	<0.001
TAG_OA:PUFA	0.97 (0.90–1.14)	1.42 (1.10–1.62)	0.006	1.98 (1.72–2.22)	<0.001

*Results are presented as median (P25–P75). CE, cholesteryl esters; DAG, diacylglycerols; FFA, free fatty acids; LPC, lysophosphatidylcholines; LPE, lysophosphatidylethanolamines; PC, phosphatidylcholines; PE, phosphatidylethanolamines; TAG, triacylglycerols; PA, palmitic acid; OA, oleic acid; SFA, saturated fatty acid; MUFA, monounsaturated fatty acid; PUFA, polyunsaturated fatty acid*.

Taken together, these data indicate a rise of PA and OA plasma levels upon intake of high levels of these FA. A slight PA enrichment in diet already induces a drop of PUFA, including linoleic acid in major lipid classes. Elevation of plasma SFA and MUFA ratios to PUFA was also observed within several lipid classes in rabbit fed both low and high lard-enriched diets.

### Correlations Between Calcification, Lipid Ratios, and Circulating Biomarkers

To get insight into potential mechanisms of diet-induced vascular and valvular calcification, we performed correlation analyses between modified lipids, biomarkers and extent of calcification in aorta and aortic valves. We first searched for correlations between aorta or aortic valve calcification and biomarkers. Among biomarkers that were significantly influenced over time by the diets, we found that the monocyte count was negatively correlated with aortic valve calcification in the 5% lard group (*r* = −0.89, *P* = 0.018). We then analyzed the correlations between the extent of calcification and lipids. These analyses revealed several correlations in the 5% lard group. Aortic valve calcification was positively correlated with ratios of PA or SFA to PUFA in DAG and TAG (PA/PUFA in DAG, ρ = 0.83, *P* = 0.042; SFA/PUFA with 18C-long FA chain in DAG, ρ = 0.89, *P* = 0.019, and in TAG, ρ = 0.91, *P* = 0.013; PA/PUFA with 18C-long FA chain FA in DAG, ρ = 0.82, *P* = 0.047 and in TAG, ρ = 0.84, *P* = 0.038). Since the drop of monocyte count observed in the 5% lard group correlated with aortic valve calcification, we then wanted to assess the relationship between this biomarker and the identified lipid ratios. Interestingly, negative correlations were observed between monocyte count and PA/PUFA ratio in DAG (*r* = −0.9, *P* = 0.014) and PA/PUFA with 18C-long FA chain in DAG (*r* = −0.87, *P* = 0.026).

PA content in total lipids and SFA content in CE were positively correlated with aorta calcification (ρ = 0.83, *P* = 0.042; ρ = 0.89, *P* = 0.019). Aorta calcification also showed positive correlations with SFA/PUFA ratio in PE (ρ = 0.89, *P* = 0.019), and OA/PUFA ratio in FFA (ρ = 0.83, *P* = 0.042). We found that PA content in total lipids positively correlated with the hematocrit (ρ = 0.83, *P* = 0.042) that was found to be elevated in the 5% lard group.

## Discussion

**Figure 4:**
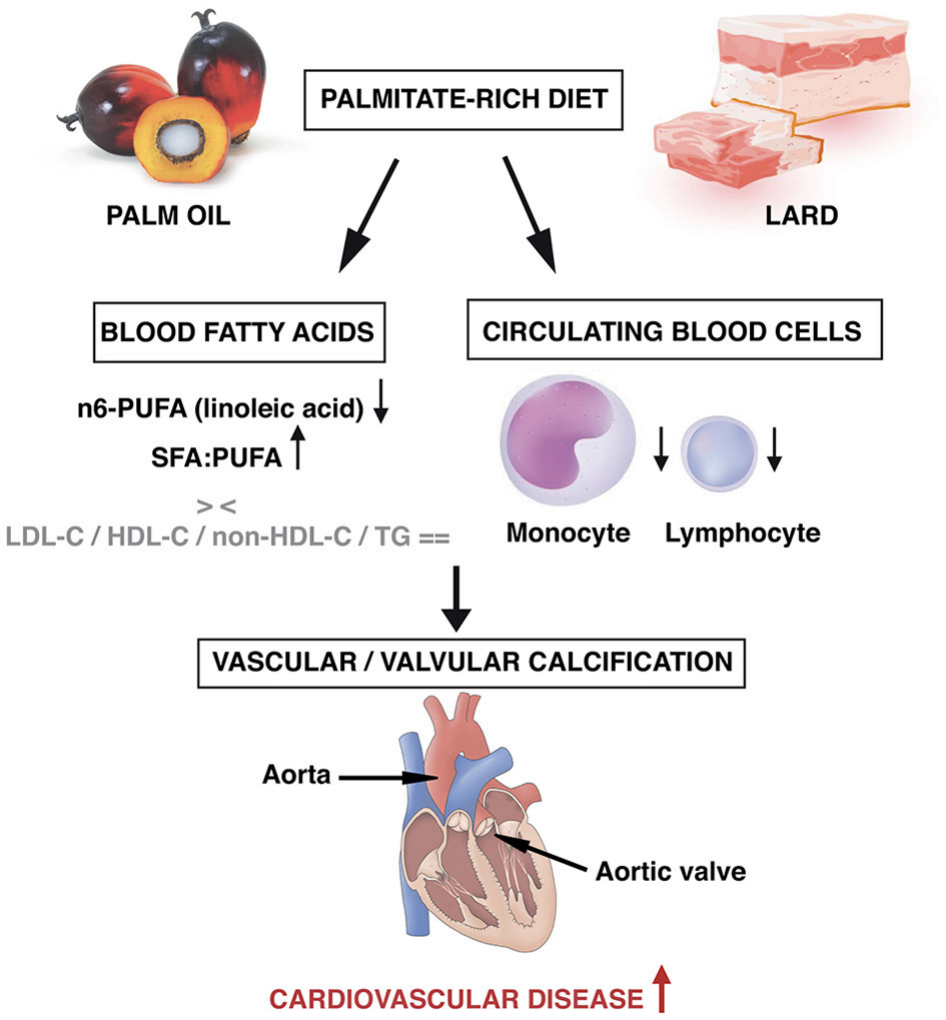
Intake of regular amounts of palmitate, a major dietary saturated fatty acid contained in palm oil and in lard, may promote calcification in aorta and in aortic valve, responsible for an increased burden of cardiovascular disease. In blood, decreased levels of polyunsaturated fatty acids as well as signs of low-grade inflammation may occur, without any changes in traditional cholesterol parameters.

Our study is the first experimental demonstration that regular dietary PA intake causes aorta and aortic valve calcification (Figure 4). Rabbits fed a diet slightly enriched in PA (0.175% of lard) for a so short period as 16 weeks developed microcalcifications both in aorta and aortic valve. Surprisingly, the extent of calcification was similar to that obtained with a higher PA food content and OA enrichment (5% of lard). This might be related to protective effects of OA (23) that might counteract the additive effects of high PA content on the calcification process.

Both diets led to a drop of plasma PUFA within the most abundant lipid classes, in line with reported beneficial effects of PUFA in cardiovascular prevention (24). Of particular interest is the decrease in linoleic acid content in total lipids and in three major lipid classes (CE, TAG, DAG). Indeed, intake of this n-6 PUFA has been associated with a decreased CAD risk (25, 26).

We did not find any modification of traditional cholesterol parameters by the two diets, excluding typical LDL-C or non-HDL-C related effects. Importantly, our findings could, at least partly, explain why a significant part of patients with CVD have normal values of cholesterol parameters (27, 28). Hence, dietary PA intake and subsequent changes in plasma SFA and PUFA might promote CVD independently of traditional risk factors.

In line with this proposition, the Bruneck lipidomic study identified levels of individual species from CE and PE as a molecular signature for CVD risk (3). Also, a recent review of several studies indicated that lipid content in LDL and HDL independent of cholesterol could play a key role as biomarkers of CVD outcomes (29).

Interestingly, we found that the extent of aorta calcification was positively correlated with SFA levels in cholesteryl esters and SFA/PUFA ratio in phosphatidylethanolamines, while SFA and PA to PUFA ratios in DAG and TAG were positively correlated with aortic valve calcification. These data suggest that the high cardiovascular risk that associated with PA could be related to pro-calcifying effects on arteries or aortic valve. Indeed, the extent of intimal vascular calcification that accompanies atherosclerosis progression has been associated with adverse cardiovascular events (30). Likewise, in calcific aortic stenosis, aortic valve calcification predicts disease severity and poor clinical outcomes (31). The study by Torzewski et al. (32) indicates that PA and OA composed the most abundant lysophosphatidic acid species in calcified valve leaflets. Their levels were highly increased in aortic valve leaflets with large calcified nodules, suggesting a possible role for these two fatty acids in aortic valve calcification. Also, in atherosclerosis (33) and in calcific aortic stenosis (34), calcium deposits in tissues at a (sub)micrometer scale (microcalcification) represent early pathological steps. It is therefore tempting to speculate that PA intake might both contribute to disease initiation and to progression.

We found that the 0.175% lard diet induced a drop of lymphocyte and monocyte counts, with concomitant increase of neutrophil percentages and NLR. We did not, however, detect any increase of the inflammatory marker IL-6. This result might indicate that regular PA intake may result in the establishment of a low-grade inflammation, a risk factor for CVD, even at very low LDL-C (35). Several *in vitro* studies provide evidence for a link between SFA and inflammation, through direct effects on monocytes/macrophages. For instance, long-chain SFA have been shown to induce the expression of pro-inflammatory cytokines in macrophages (36). It has also been reported that SFA promotes the expression of GDF-15 in human macrophages (37), a marker of poor outcome both in CAD (38) and calcific aortic stenosis (39).

The observed drop in monocyte count could be due to higher recruitment of blood monocytes in vascular or valvular tissues and their subsequent differentiation into macrophages, a key event in the initiation of the calcification process (40). The fact that monocyte counts were negatively correlated with both aortic valve calcification and PA/PUFA ratios in DAG might reveal novel mechanistic clues, which warrants further investigations. PA could either promote aortic valve calcification through macrophage activation or differentiation (41) or, indirectly, via a PUFA-lowering effect (42).

We found that PA content in total lipids correlated with aorta calcification. Our observation that lard diets induced a rise of haematocrit that positively correlated with PA content in total lipids would therefore suggest the existence of a link between haematocrit levels and aorta calcification following PA intake. To date, there is no clear evidence for an association between haematocrit and CAD (43–46) although some studies indicate that a rise of hematocrit and subsequent blood viscosity may increase endothelial shear stress and contribute to the initiation and progression of atherosclerosis (47).

## Limitations

Our study has some limitations, including low numbers of rabbits, which did not allow us reaching high statistical power. Only male rabbits were included. Also, we did not provide functional demonstration of causal relationship between plasma lipid changes and vascular or valvular calcification. Further investigations are needed to establish the link between PA intake, changes in circulating leukocytes and hematocrit levels and tissue calcification. Also, whether FA, free or in lipids, can serve as sites of calcium precipitation in vascular or valvular tissues remains to be determined. The alizarin red staining enabled us to detect small calcium deposits. Nevertheless, this method did not allow us to visualize crystal shape or structures, which might inform on mineralization mechanisms.

## Conclusions

Our study showed that a regular intake of PA-rich lard promotes aorta and aortic valve calcification through plasma lipid modification independent of conventional cholesterol markers. PA is also a main component of palm oil and, as such, it can form a large proportion of total dietary SFA intake. Our study therefore calls for caution when consuming too much of PA-containing food on a regular basis. We propose to consider using both PA and PUFA levels as markers of CV health.

## Data Availability Statement

The original contributions presented in the study are included in the article/Supplementary Material, further inquiries can be directed to the corresponding authors.

## Ethics Statement

The animal study was reviewed and approved by Animal Ethical Committee of the University of Liège.

## Author Contributions

CO, PL, and AN contributed to conception and design of the study. ZJ performed the statistical analysis. ND and CO wrote the manuscript. CD'E, NB, RD, and ND performed the experiments. MG and PD contributed to data interpretation. All authors contributed to manuscript revision, read, and approved the submitted version.

## Conflict of Interest

The authors declare that the research was conducted in the absence of any commercial or financial relationships that could be construed as a potential conflict of interest.
