# Supplementary material for: Regular Dietary Intake of Palmitate Causes Vascular and Valvular Calcification in a Rabbit Model
Source: Front Cardiovasc Med. 2021 Jun 23;8:692184. doi: 10.3389/fcvm.2021.692184 (PMC8261064; doi:10.3389/fcvm.2021.692184)
Supplement: Supplementary file 1 [file Data_Sheet_1.PDF]

Regular dietary intake of palmitate causes vascular and valvular calcification in a rabbit model

– *Supplementary data*

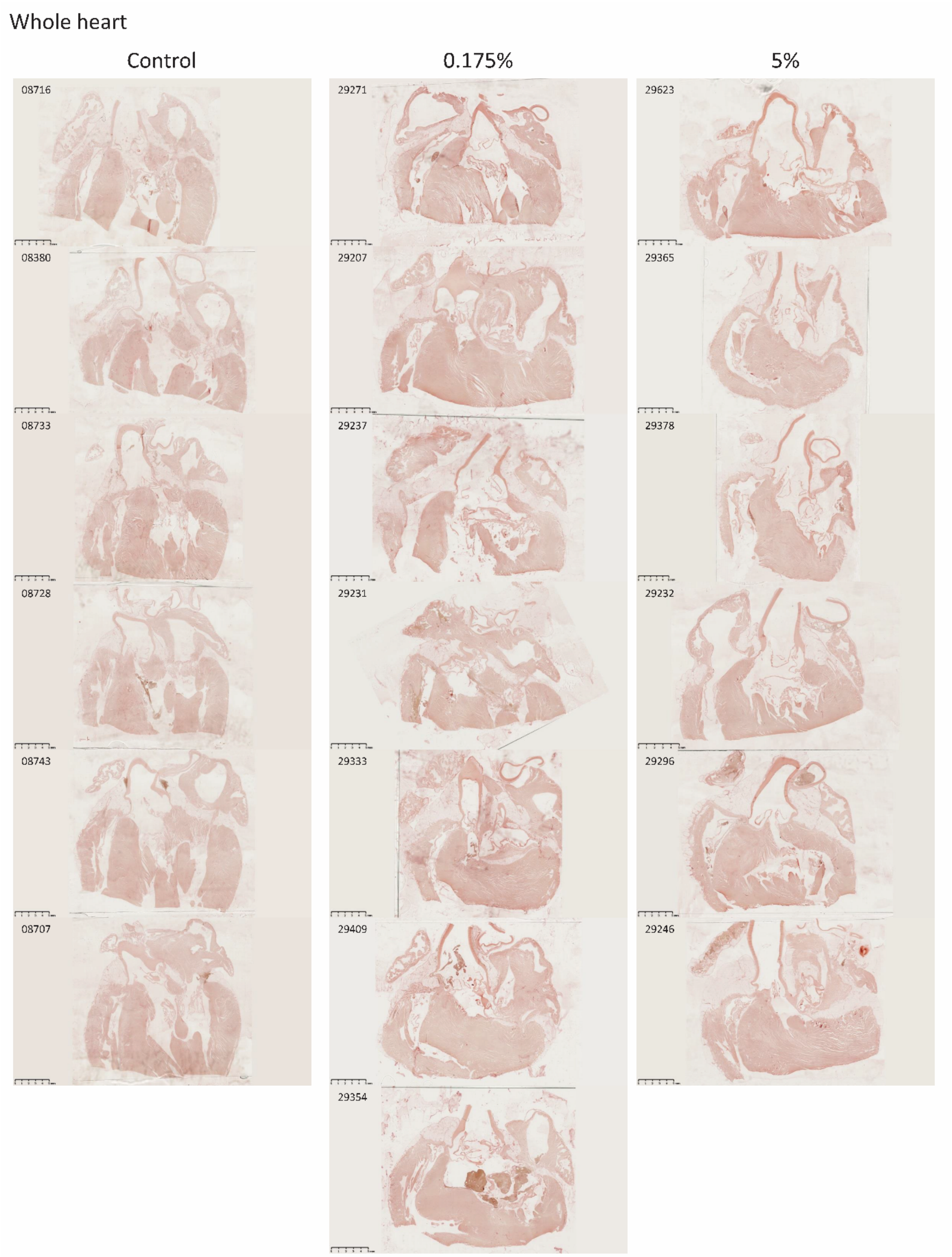

Myocardium

Control	0.175%	5%

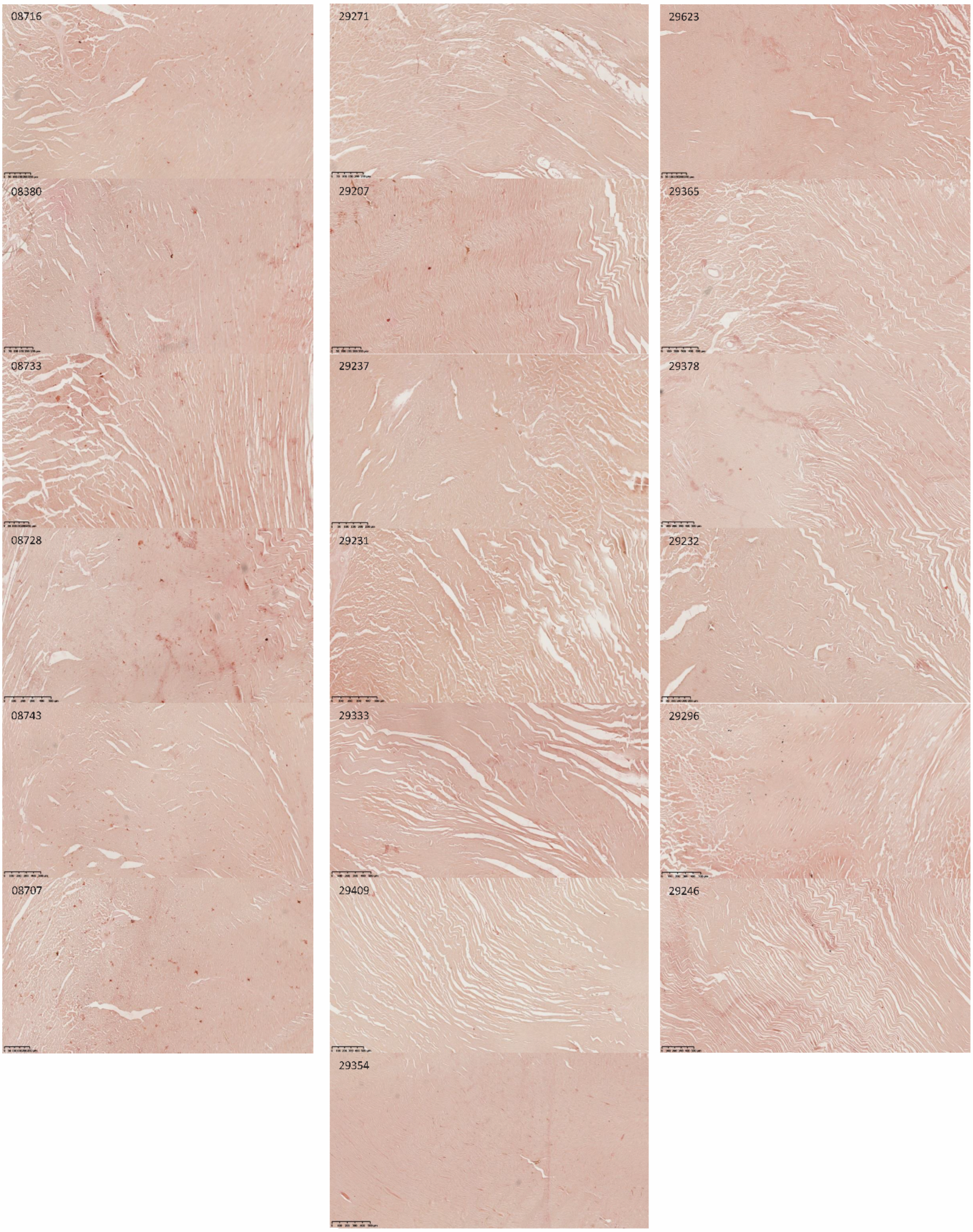

Aorta & Aortic valve_large view

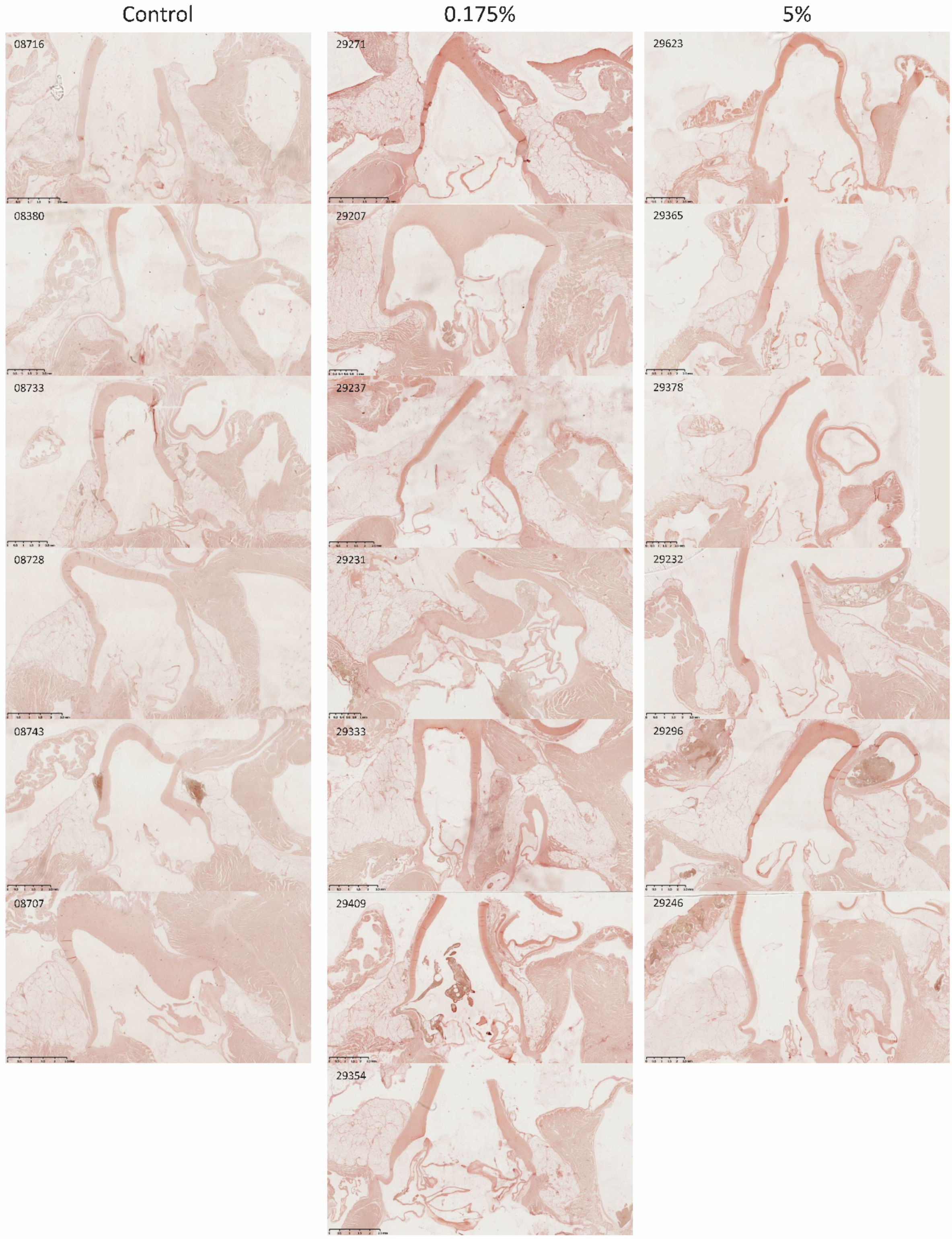

Aorta_close view

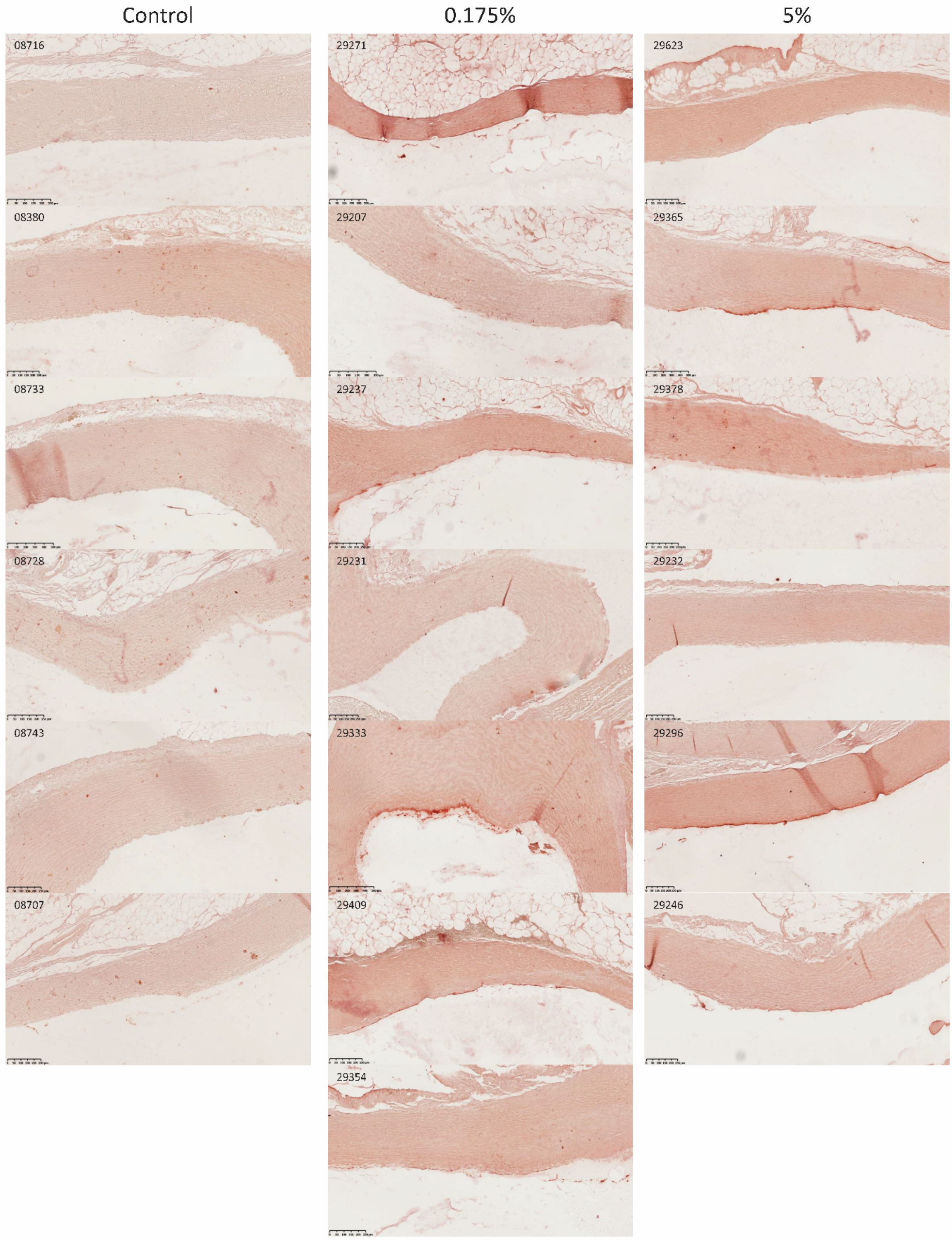

Aortic valve_large view

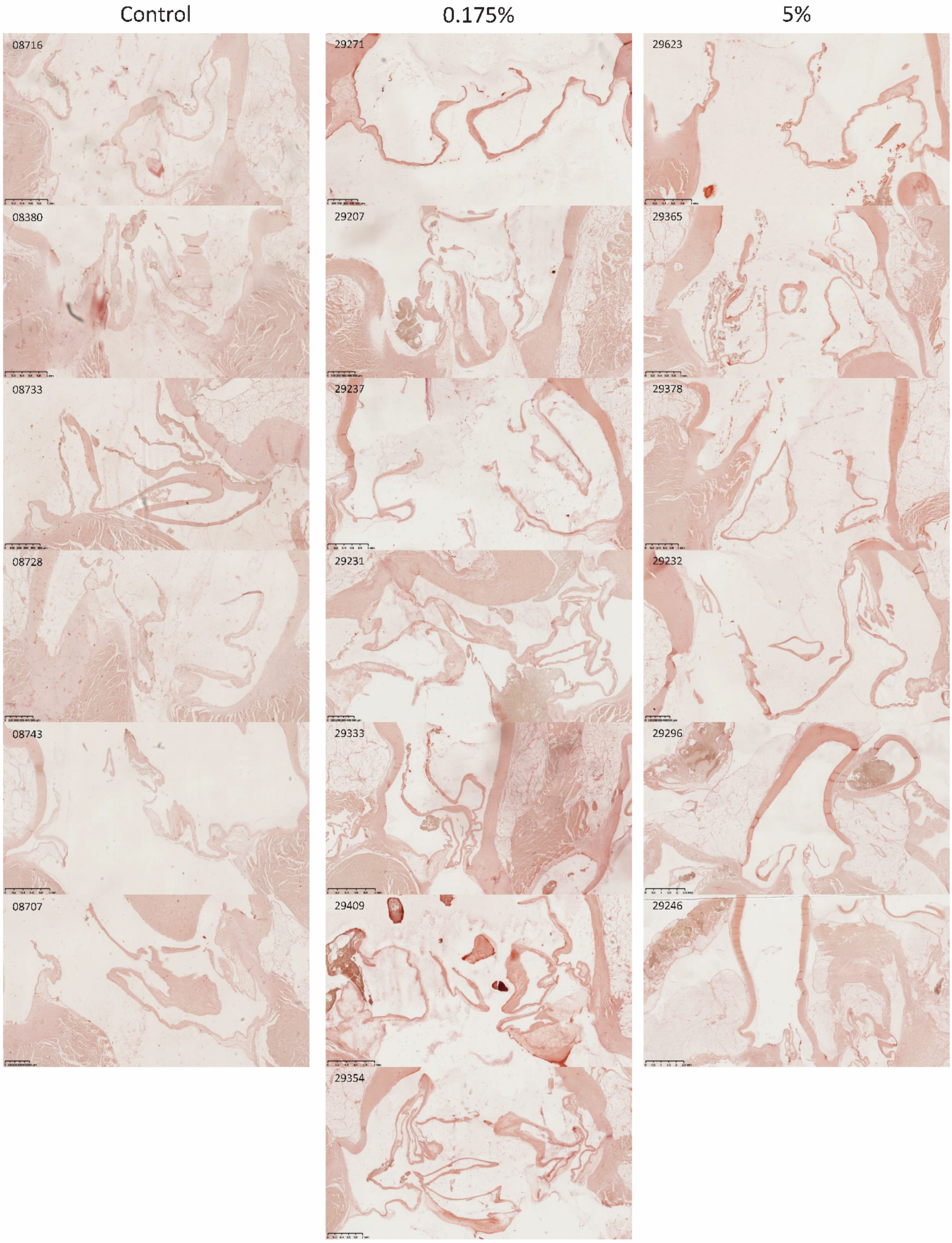

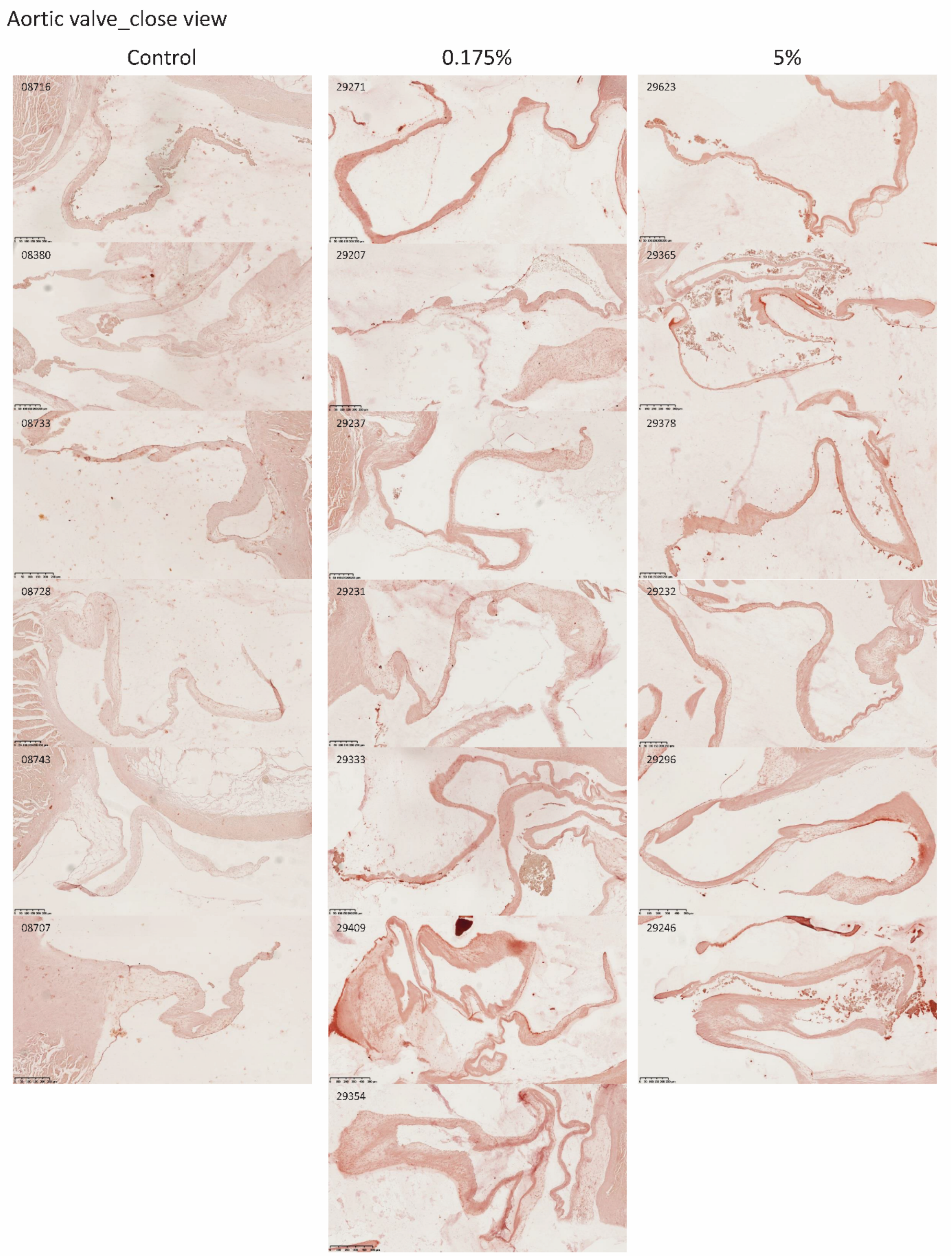

Figure S1. Pictures of alizarin red staining on histological hearts sections from each rabbit in all three diet groups.

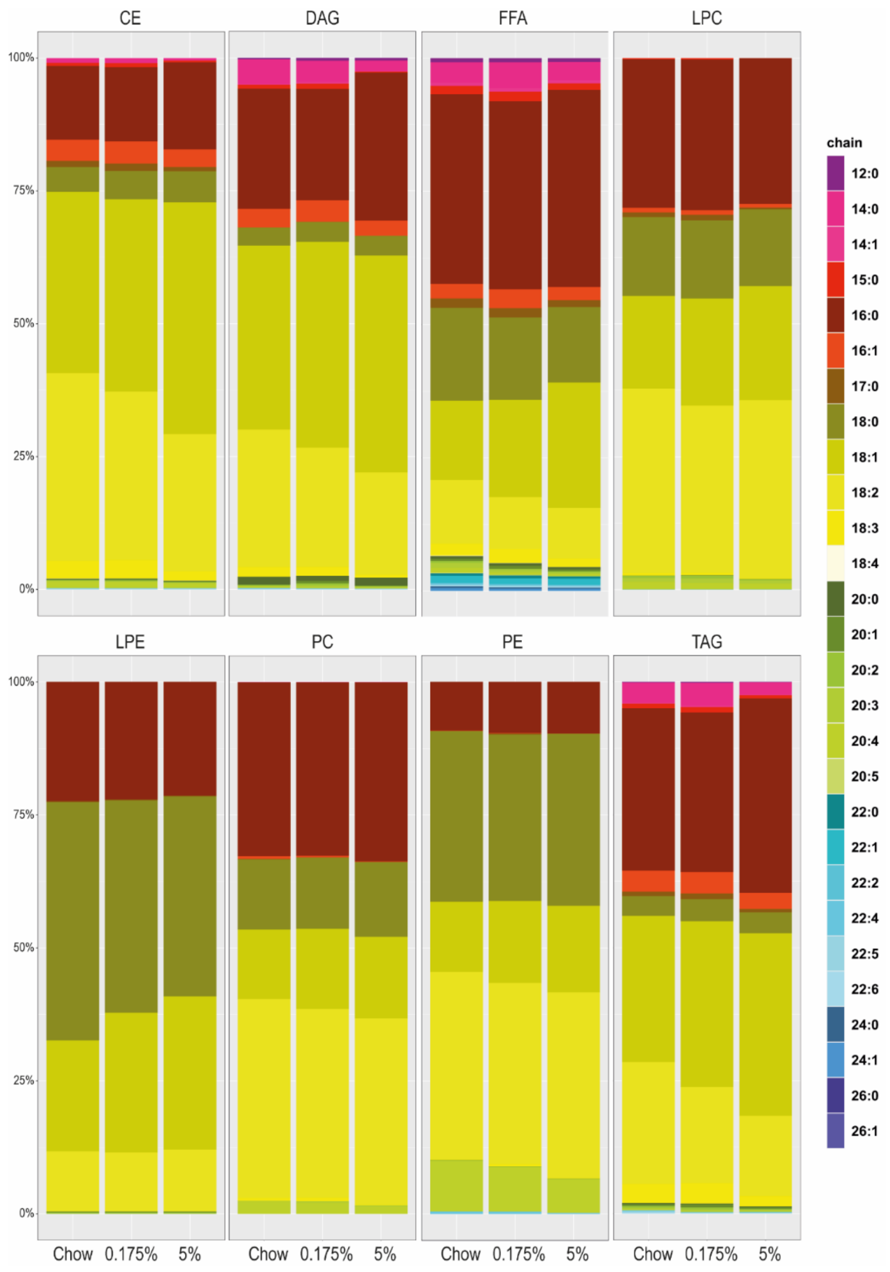

Figure S2. Representation of fatty acid percentages in lipid classes. CE : cholesteryl esters. DAG : diacylglycerols. FFA : free fatty acids. LPC : Lysophosphatidylcholines. LPE : lysophosphatidylethanolamines. PC : phosphatidylcholines. PE : phosphatidylethanolamines. SM : sphingomyelins. TAG : triacylglycerols.

Table S2. Comparisons of lipid class concentrations and percentages, lipid category percentages and lipid concentration ratios between 0.175% or 5% groups and chow group.

	Chow	0.175%	*P-value* vs chow	5%	*P-value* vs chow
Lipid class concentration (nmol/g)					
CE	259.54 (177.50-283.19)	202.92 (153.42-291.48)	0.970	236.32 (187.47-291.68)	0.998
CER	2.55 (2.29-4.72)	3.44 (2.32-5.56)	0.497	2.76 (2.22-3.24)	0.987
DAG	16.22 (10.86-23.43)	31.80 (14.89-37.64)	0.346	7.32 (5.70-34.73)	0.485
DCER	0.26 (0.25-0.31)	0.28 (0.24-0.34)	0.949	0.34 (0.23-0.38)	1.000
FFA	204.08 (105.40-239.67)	234.22 (201.83-256.68)	0.317	227.63 (178.35-299.42)	0.194
HCER	0.27 (0.19-0.35)	0.21 (0.10-0.34)	0.801	0.19 (0.17-0.20)	0.708
LCER	0.09 (0.01-0.15)	0.28 (0.07-0.34)	0.152	0.05 (0.01-0.09)	0.434
LPC	243.55 (219.92-268.39)	223.31 (208.33-304.76)	0.938	193.88 (164.87-210.10)	0.018
LPE	2.86 (2.53-3.04)	2.56 (2.45-3.36)	0.906	2.27 (1.84-2.80)	0.257
PC	332.91 (295.12-431.03)	452.90 (298.86-602.39)	0.553	260.21 (172.44-378.28)	0.659
PE	56.01 (33.70-82.31)	78.39 (50.51-82.94)	0.574	30.85 (20.38-58.42)	0.230
SM	216.85 (177.80-261.05)	267.58 (205.26-399.31)	0.520	175.73 (123.79-219.40)	0.520
TAG	853.27 (597.33-1679.05)	1562.06 (719.71-1854.32)	0.588	288.94 (257.77-1294.09)	0.167
Lipid classes (%)					
CE	10.00 (8.19-11.72)	8.24 (6.26-8.98)	0.147	13.13 (8.73-16.69)	0.237
CER	0.13 (0.12-0.15)	0.13 (0.11-0.15)	0.998	0.17 (0.13-0.18)	0.135
DAG	0.69 (0.68-0.80)	0.98 (0.69-1.07)	0.333	0.54 (0.51-0.99)	0.603
DCER	0.01 (0.01-0.02)	0.01 (0.01-0.01)	0.325	0.01 (0.01-0.03)	0.492
FFA	7.49 (5.44-10.06)	7.36 (5.40-14.39)	0.952	16.89 (9.72-18.43)	0.101
HCER	0.01 (0.01-0.01)	0.01 (0.00-0.01)	0.129	0.01 (0.01-0.01)	0.999
LCER	0.00 (0.00-0.01)	0.01 (0.00-0.01)	0.239	0.00 (0.00-0.01)	0.472
LPC	11.11 (7.41-15.31)	8.88 (6.75-9.27)	0.319	10.52 (7.37-14.68)	0.957
LPE	0.12 (0.10-0.14)	0.10 (0.08-0.11)	0.465	0.13 (0.10-0.17)	0.989
PC	13.78 (13.36-17.40)	14.98 (12.98-15.92)	0.993	15.12 (14.14-15.90)	0.808
PE	2.39 (2.36-2.54)	2.42 (2.30-2.56)	0.986	2.22 (1.81-2.29)	0.195
SM	10.52 (8.06-12.71)	10.39 (7.86-11.82)	0.985	10.34 (8.29-11.35)	0.996

TAG	43.18 (29.95-48.61)	47.89 (34.57-53.16)	0.814	23.74 (19.08-48.38)	0.069
SFA-MUFA-PUFA contents (%)					
SFA	46.94 (43.13-48.05)	47.82 (46.59-48.28)	0.535	49.06 (48.53-49.42)	0.060
MUFA	25.60 (20.80-27.19)	28.71 (24.74-29.51)	0.147	29.99 (27.43-30.77)	0.046
PUFA	28.55 (27.37-30.67)	23.93 (23.06-25.78)	0.002	21.44 (21.07-23.25)	<0.001
CE_SFA	20.67 (20.18-22.39)	21.93 (21.24-24.31)	0.311	23.91 (23.32-24.49)	0.018
CE_MUFA	35.96 (32.21-45.54)	43.92 (33.20-44.84)	0.756	48.15 (47.27-49.09)	0.077
CE_PUFA	41.65 (34.28-46.34)	35.32 (33.72-42.50)	0.521	27.29 (26.98-29.42)	0.008
DAG_SFA	33.95 (29.16-35.52)	31.50 (29.20-33.24)	0.462	36.55 (34.84-36.98)	0.266
DAG_MUFA	36.72 (35.91-40.89)	43.70 (40.70-48.34)	0.027	43.88 (40.10-46.37)	0.024
DAG_PUFA	28.86 (26.02-30.24)	23.88 (23.06-27.20)	0.092	20.05 (18.50-22.08)	<0.001
FFA_SFA	60.32 (58.95-68.87)	62.70 (57.14-64.17)	0.955	59.77 (58.04-61.81)	0.576
FFA_MUFA	20.01 (15.59-23.50)	22.47 (21.41-28.54)	0.096	28.18 (26.33-28.74)	0.004
FFA_PUFA	17.52 (15.54-19.95)	14.32 (13.48-16.16)	0.072	12.45 (11.56-13.33)	0.005
LPC_SFA	43.18 (39.24-46.81)	44.11 (42.24-46.00)	0.933	41.56 (39.98-44.26)	0.685
LPC_MUFA	18.55 (16.10-20.69)	22.67 (18.50-22.94)	0.055	22.31 (21.30-23.32)	0.008
LPC_PUFA	38.68 (37.14-41.12)	34.10 (32.37-36.60)	0.204	36.16 (35.19-37.08)	0.503
LPE_SFA	65.82 (63.09-69.87)	61.78 (58.71-68.22)	0.448	54.87 (53.15-57.61)	0.129
LPE_MUFA	20.38 (18.18-24.72)	26.01 (22.28-29.94)	0.099	31.94 (28.42-33.19)	0.031
LPE_PUFA	12.54 (10.75-15.22)	10.50 (7.83-15.45)	0.862	14.48 (11.84-14.71)	1.000
PC_SFA	46.03 (45.02-47.06)	45.84 (44.88-47.28)	1.000	47.38 (46.15-48.59)	0.199
PC_MUFA	14.24 (10.69-15.29)	16.30 (12.65-18.28)	0.263	15.53 (14.89-16.14)	0.214
PC_PUFA	40.94 (38.81-41.81)	38.52 (36.86-39.80)	0.294	37.52 (36.20-38.20)	0.030
PE_SFA	41.14 (40.86-42.17)	41.06 (40.03-41.90)	0.915	42.24 (41.71-43.37)	0.312
PE_MUFA	13.66 (12.55-14.16)	16.02 (13.34-17.97)	0.061	15.46 (15.00-16.79)	0.015
PE_PUFA	45.45 (45.03-46.08)	43.22 (42.12-44.93)	0.012	41.75 (40.80-42.78)	<0.001
TAG_SFA	40.09 (36.97-43.27)	40.96 (38.42-42.64)	0.718	44.45 (43.00-45.62)	0.008
TAG_MUFA	31.88 (28.93-35.73)	35.96 (32.24-40.29)	0.061	38.12 (35.50-40.25)	0.012
TAG_PUFA	27.80 (26.29-30.70)	21.66 (21.02-25.58)	0.004	17.72 (16.37-18.88)	<0.001
FA content (% of total FA)					
FA(12:0)	0.12 (0.11-0.15)	0.14 (0.14-0.15)	0.185	0.13 (0.09-0.14)	0.642

FA(14:0)	2.89 (1.87-3.00)	2.92 (2.77-3.59)	0.188	1.52 (1.09-1.73)	0.019
FA(14:1)	0.16 (0.13-0.22)	0.26 (0.14-0.30)	0.248	0.12 (0.08-0.18)	0.356
FA(15:0)	0.68 (0.59-0.70)	0.82 (0.59-0.97)	0.044	0.43 (0.40-0.53)	0.022
PA - FA(16:0)	28.89 (26.48-29.07)	27.70 (26.96-29.06)	0.996	30.31 (29.63-32.12)	0.005
FA(16:1)	2.95 (2.28-3.60)	3.24 (2.60-3.66)	0.834	2.06 (1.67-2.46)	0.046
FA(17:0)	0.72 (0.63-0.75)	0.86 (0.74-0.93)	0.003	0.50 (0.45-0.56)	<0.001
FA(18:0)	6.68 (6.26-7.85)	6.61 (6.20-8.36)	0.979	8.66 (6.36-9.50)	0.110
OA - FA(18:1)	23.42 (19.70-24.59)	26.52 (22.81-27.26)	0.089	27.14 (26.05-29.89)	0.018
FA(18:2)	24.81 (23.64-26.88)	19.56 (19.41-22.43)	<0.001	18.92 (18.24-21.04)	<0.001
FA(18:3)	2.70 (2.08-3.09)	2.87 (2.84-3.05)	0.479	1.17 (1.10-1.53)	<0.001
FA(18:4)	0.00 (0.00-0.01)	0.00 (0.00-0.01)	0.935	0.01 (0.00-0.01)	0.242
FA(20:0)	1.66 (1.11-2.11)	1.57 (1.15-1.88)	0.683	1.57 (1.50-1.80)	0.991
FA(20:1)	0.30 (0.24-0.34)	0.48 (0.40-0.59)	0.007	0.25 (0.22-0.30)	0.894
FA(20:2)	0.24 (0.22-0.28)	0.30 (0.25-0.41)	0.091	0.14 (0.12-0.23)	0.113
FA(20:3)	0.19 (0.18-0.20)	0.27 (0.24-0.34)	0.033	0.16 (0.12-0.24)	0.680
FA(20:4)	0.87 (0.85-0.95)	0.74 (0.52-0.93)	0.288	0.54 (0.46-0.93)	0.128
FA(20:5)	0.03 (0.02-0.03)	0.03 (0.02-0.03)	0.435	0.02 (0.02-0.03)	0.467
FA(22:0)	2.04 (1.90-2.83)	2.27 (1.79-2.62)	1.000	2.79 (2.10-3.02)	0.321
FA(22:1)	0.38 (0.31-0.48)	0.41 (0.35-0.48)	0.948	0.49 (0.37-0.59)	0.261
FA(22:2)	0.01 (0.01-0.01)	0.01 (0.00-0.01)	0.967	0.02 (0.00-0.02)	0.227
FA(22:4)	0.08 (0.05-0.10)	0.07 (0.06-0.08)	0.392	0.05 (0.05-0.05)	0.036
FA(22:5)	0.08 (0.08-0.11)	0.09 (0.08-0.11)	0.434	0.08 (0.07-0.10)	0.456
FA(22:6)	0.04 (0.03-0.04)	0.03 (0.03-0.04)	0.402	0.04 (0.03-0.04)	0.510
FA(24:0)	0.55 (0.45-0.91)	0.42 (0.33-0.82)	0.840	1.06 (0.39-1.21)	0.253
FA(24:1)	0.13 (0.11-0.17)	0.14 (0.12-0.16)	0.955	0.22 (0.10-0.27)	0.112
FA(26:0)	0.00 (0.00-0.00)	0.00 (0.00-0.00)	0.338	0.00 (0.00-0.00)	0.780
FA(26:1)	0.00 (0.00-0.01)	0.00 (0.00-0.00)	0.993	0.00 (0.00-0.01)	0.581
FA content in classes (%)					
CE_FA(14:0)	0.76 (0.68-1.03)	0.92 (0.81-0.99)	0.567	0.43 (0.33-0.45)	<0.001
CE_FA(14:1)	0.06 (0.05-0.08)	0.07 (0.05-0.11)	0.706	0.03 (0.02-0.04)	0.032
CE_FA(15:0)	0.60 (0.46-0.80)	0.76 (0.47-0.98)	0.428	0.39 (0.32-0.42)	0.050
CE_FA(16:0)	14.22 (12.25-14.98)	13.83 (12.41-15.55)	0.992	16.22 (15.79-17.48)	0.030
CE_FA(16:1)	3.76 (3.12-4.40)	4.36 (3.58-4.92)	0.847	3.34 (3.06-3.82)	0.291

CE_FA(17:0)	1.13 (1.00-1.21)	1.38 (1.24-1.55)	0.094	0.79 (0.70-0.95)	0.009
CE_FA(18:0)	4.84 (3.50-5.24)	5.77 (4.56-6.05)	0.416	5.93 (5.62-6.18)	0.084
CE_FA(18:1)	32.78 (28.29-40.91)	39.10 (29.45-39.21)	0.770	44.84 (44.82-45.08)	0.036
CE_FA(18:2)	36.22 (29.60-41.26)	30.67 (28.74-36.95)	0.481	24.15 (23.71-26.46)	0.021
CE_FA(18:3)	3.39 (3.20-3.42)	3.38 (3.03-3.65)	0.826	1.77 (1.65-1.88)	<0.001
CE_FA(18:4)	0.03 (0.03-0.04)	0.03 (0.03-0.04)	0.615	0.03 (0.02-0.03)	0.430
CE_FA(20:0)	0.07 (0.06-0.08)	0.09 (0.08-0.10)	0.057	0.07 (0.05-0.09)	0.937
CE_FA(20:1)	0.14 (0.10-0.24)	0.24 (0.15-0.36)	0.165	0.14 (0.10-0.20)	0.998
CE_FA(20:2)	0.11 (0.07-0.15)	0.13 (0.12-0.18)	0.446	0.07 (0.06-0.08)	0.139
CE_FA(20:3)	0.16 (0.16-0.17)	0.18 (0.18-0.19)	0.363	0.12 (0.11-0.14)	0.019
CE_FA(20:4)	1.26 (0.86-1.55)	1.11 (0.91-1.33)	0.840	0.84 (0.68-1.08)	0.156
CE_FA(20:5)	0.04 (0.03-0.05)	0.06 (0.05-0.07)	0.052	0.04 (0.02-0.04)	0.550
CE_FA(22:0)	0.00 (0.00-0.03)	0.00 (0.00-0.03)	0.994	0.00 (0.00-0.02)	0.928
CE_FA(22:1)	0.00 (0.00-0.02)	0.00 (0.00-0.03)	0.992	0.00 (0.00-0.00)	0.893
CE_FA(22:2)	0.00 (0.00-0.00)	0.00 (0.00-0.00)	1.000	0.00 (0.00-0.00)	1.000
CE_FA(22:4)	0.03 (0.02-0.03)	0.00 (0.00-0.02)	0.036	0.02 (0.00-0.02)	0.370
CE_FA(22:5)	0.09 (0.07-0.11)	0.09 (0.08-0.10)	0.977	0.10 (0.08-0.11)	0.821
CE_FA(22:6)	0.12 (0.07-0.14)	0.12 (0.09-0.13)	0.982	0.10 (0.07-0.13)	0.962
CE_FA(24:0)	0.00 (0.00-0.00)	0.00 (0.00-0.00)	0.406	0.00 (0.00-0.00)	0.432
CE_FA(24:1)	0.00 (0.00-0.00)	0.00 (0.00-0.00)	1.000	0.00 (0.00-0.00)	1.000
DAG_FA(12:0)	0.28 (0.01-0.51)	0.54 (0.22-1.00)	0.589	0.27 (0.01-0.35)	1.000
DAG_FA(14:0)	3.58 (3.41-4.15)	3.70 (3.39-4.54)	0.978	2.00 (1.71-2.59)	0.007
DAG_FA(14:1)	0.01 (0.01-0.11)	0.38 (0.15-0.49)	0.043	0.01 (0.01-0.01)	0.794
DAG_FA(15:0)	0.68 (0.44-1.09)	1.03 (0.73-1.20)	0.746	0.32 (0.02-0.43)	0.016
DAG_FA(16:0)	24.56 (19.45-25.11)	21.16 (19.00-23.17)	0.430	28.66 (27.22-28.92)	0.007
DAG_FA(16:1)	3.08 (3.04-4.07)	3.91 (3.39-4.95)	0.427	2.85 (2.44-3.53)	0.273
DAG_FA(18:0)	3.53 (3.09-3.58)	3.52 (3.40-3.97)	0.493	3.75 (3.55-4.25)	0.565
DAG_FA(18:1)	33.61 (32.68-36.46)	37.94 (36.82-41.07)	0.029	41.49 (37.47-42.81)	0.003
DAG_FA(18:2)	26.06 (23.43-27.66)	21.08 (19.88-24.67)	0.053	18.80 (17.66-20.72)	<0.001
DAG_FA(18:3)	1.82 (1.63-1.94)	1.61 (1.58-1.91)	0.965	0.64 (0.53-0.74)	<0.001
DAG_FA(20:0)	1.28 (0.96-2.02)	0.79 (0.63-1.15)	0.324	1.39 (0.73-2.06)	0.996
DAG_FA(20:1)	0.02 (0.02-0.22)	0.45 (0.33-0.59)	0.006	0.02 (0.02-0.23)	0.994
DAG_FA(20:2)	0.02 (0.02-0.32)	0.38 (0.29-0.53)	0.106	0.02 (0.02-0.02)	0.492

DAG_FA(20:3)	0.02 (0.02-0.19)	0.39 (0.28-0.44)	0.014	0.09 (0.02-0.23)	0.851
DAG_FA(20:4)	0.26 (0.16-0.40)	0.21 (0.17-0.27)	0.988	0.17 (0.01-0.30)	0.408
DAG_FA(20:5)	0.02 (0.02-0.02)	0.02 (0.02-0.02)	1.000	0.02 (0.02-0.02)	1.000
DAG_FA(22:4)	0.17 (0.01-0.20)	0.12 (0.01-0.22)	0.999	0.04 (0.01-0.08)	0.579
DAG_FA(22:5)	0.01 (0.01-0.01)	0.01 (0.01-0.01)	0.866	0.01 (0.01-0.01)	0.915
DAG_FA(22:6)	0.05 (0.05-0.05)	0.05 (0.05-0.05)	1.000	0.05 (0.05-0.05)	1.000
FFA(_FA(12:0)	0.70 (0.63-0.95)	0.77 (0.60-0.80)	0.973	0.59 (0.39-0.94)	0.546
FFA(_FA(14:0)	3.83 (3.52-4.23)	4.49 (4.03-5.33)	0.119	3.44 (2.91-3.83)	0.599
FFA(_FA(14:1)	0.61 (0.53-0.65)	0.73 (0.54-0.84)	0.644	0.48 (0.40-0.63)	0.253
FFA(_FA(15:0)	1.66 (1.27-1.83)	1.78 (1.19-2.32)	0.607	1.22 (1.04-1.43)	0.276
FFA(_FA(16:0)	33.54 (33.34-39.70)	35.87 (34.12-36.31)	0.988	36.55 (35.44-36.92)	0.449
FFA(_FA(16:1)	2.68 (2.18-3.23)	3.55 (2.86-4.11)	0.086	2.46 (2.17-2.98)	0.593
FFA(_FA(17:0)	1.59 (1.35-2.35)	1.69 (1.49-1.98)	0.973	1.35 (1.10-1.44)	0.059
FFA(_FA(18:0)	17.09 (16.57-18.71)	14.68 (12.74-17.46)	0.438	15.04 (12.79-15.83)	0.186
FFA(_FA(18:1)	14.69 (10.52-18.68)	16.54 (15.39-22.16)	0.127	23.20 (21.84-24.22)	0.002
FFA(_FA(18:2)	12.50 (9.56-13.41)	9.74 (9.06-11.18)	0.151	9.53 (8.53-10.19)	0.127
FFA(_FA(18:3)	2.22 (1.70-2.75)	2.35 (2.20-2.94)	0.564	1.44 (1.34-1.74)	0.026
FFA(_FA(18:4)	0.06 (0.05-0.14)	0.08 (0.07-0.09)	0.969	0.05 (0.04-0.06)	0.161
FFA(_FA(20:0)	0.47 (0.42-0.63)	0.55 (0.43-0.58)	0.925	0.43 (0.39-0.44)	0.461
FFA(_FA(20:1)	0.37 (0.32-0.41)	0.49 (0.43-0.53)	0.331	0.38 (0.32-0.42)	0.891
FFA(_FA(20:2)	0.33 (0.31-0.42)	0.32 (0.29-0.36)	0.827	0.24 (0.19-0.28)	0.023
FFA(_FA(20:3)	0.32 (0.23-0.48)	0.40 (0.27-0.46)	0.686	0.27 (0.18-0.28)	0.176
FFA(_FA(20:4)	0.62 (0.45-1.11)	0.52 (0.38-0.58)	0.179	0.37 (0.30-0.50)	0.031
FFA(_FA(20:5)	0.12 (0.08-0.20)	0.09 (0.08-0.11)	0.301	0.07 (0.06-0.08)	0.043
FFA(_FA(22:0)	0.51 (0.40-0.59)	0.51 (0.43-0.56)	0.951	0.43 (0.39-0.46)	0.484
FFA(_FA(22:1)	1.09 (0.60-1.25)	1.20 (0.84-1.31)	0.980	1.14 (0.85-1.40)	0.998
FFA(_FA(22:2)	0.17 (0.13-0.22)	0.21 (0.19-0.25)	0.836	0.14 (0.12-0.18)	0.292
FFA(_FA(22:4)	0.10 (0.09-0.15)	0.10 (0.09-0.13)	0.778	0.08 (0.07-0.09)	0.119
FFA(_FA(22:5)	0.22 (0.17-0.25)	0.19 (0.16-0.29)	0.883	0.18 (0.13-0.19)	0.209
FFA(_FA(22:6)	0.09 (0.09-0.17)	0.11 (0.10-0.12)	0.850	0.09 (0.08-0.10)	0.321
FFA(_FA(24:0)	0.22 (0.18-0.36)	0.29 (0.22-0.31)	0.957	0.22 (0.21-0.25)	0.445
FFA(_FA(24:1)	0.42 (0.27-0.61)	0.44 (0.25-0.58)	0.769	0.44 (0.33-0.52)	0.836
LPC_FA(15:0)	0.31 (0.02-0.46)	0.23 (0.02-0.45)	0.999	0.02 (0.02-0.02)	0.071

LPC_FA(16:0)	28.33 (25.98-30.03)	28.48 (26.51-28.79)	0.904	26.71 (25.89-28.82)	0.887
LPC_FA(16:1)	0.79 (0.77-1.03)	0.89 (0.79-1.02)	0.963	0.67 (0.58-0.93)	0.181
LPC_FA(17:0)	0.83 (0.77-1.01)	0.98 (0.84-1.27)	0.903	0.19 (0.03-0.62)	0.002
LPC_FA(18:0)	14.19 (12.19-16.22)	15.24 (13.75-15.58)	0.989	14.50 (13.51-15.39)	0.985
LPC_FA(18:1)	17.51 (15.40-19.72)	21.59 (17.69-21.90)	0.046	21.50 (20.35-22.78)	0.004
LPC_FA(18:2)	35.51 (34.05-37.70)	30.59 (29.45-32.97)	0.159	34.38 (32.77-35.10)	0.836
LPC_FA(18:3)	0.51 (0.39-0.62)	0.50 (0.43-0.57)	1.000	0.04 (0.04-0.04)	0.007
LPC_FA(20:1)	0.02 (0.02-0.02)	0.02 (0.02-0.02)	1.000	0.02 (0.02-0.02)	1.000
LPC_FA(20:2)	0.45 (0.43-0.61)	0.69 (0.04-0.78)	0.982	0.04 (0.04-0.55)	0.139
LPC_FA(20:3)	0.64 (0.58-0.74)	0.86 (0.63-0.93)	0.768	0.60 (0.42-0.83)	0.410
LPC_FA(20:4)	1.50 (1.47-1.59)	1.34 (1.15-1.41)	0.087	1.08 (0.89-1.32)	0.002
LPC_FA(22:4)	0.02 (0.02-0.02)	0.02 (0.02-0.02)	1.000	0.02 (0.02-0.02)	1.000
LPC_FA(22:5)	0.01 (0.01-0.01)	0.01 (0.01-0.01)	1.000	0.01 (0.01-0.01)	1.000
LPE_FA(16:0)	22.94 (20.44-24.27)	22.07 (21.51-23.80)	0.957	20.31 (19.30-23.48)	0.638
LPE_FA(17:0)	0.18 (0.18-0.18)	0.18 (0.18-0.18)	1.000	0.18 (0.18-0.18)	1.000
LPE_FA(18:0)	45.05 (38.65-45.96)	40.11 (36.63-44.79)	0.387	33.96 (33.22-36.22)	0.112
LPE_FA(18:1)	20.16 (17.96-24.50)	25.79 (22.06-29.72)	0.099	31.72 (28.20-32.97)	0.031
LPE_FA(18:2)	12.34 (10.56-15.02)	10.31 (7.63-15.25)	0.844	14.28 (11.64-14.51)	0.999
LPE_FA(20:1)	0.22 (0.22-0.22)	0.22 (0.22-0.22)	1.000	0.22 (0.22-0.22)	1.000
LPE_FA(20:2)	0.20 (0.20-0.20)	0.20 (0.20-0.20)	1.000	0.20 (0.20-0.20)	1.000
PC_FA(14:0)	0.03 (0.03-0.03)	0.03 (0.03-0.03)	1.000	0.03 (0.03-0.03)	1.000
PC_FA(16:0)	32.74 (30.46-34.90)	30.48 (29.00-35.81)	0.992	33.96 (30.27-36.97)	0.853
PC_FA(16:1)	0.44 (0.02-0.76)	0.02 (0.02-1.08)	0.551	0.02 (0.02-0.02)	0.250
PC_FA(17:0)	0.03 (0.03-0.03)	0.03 (0.03-0.03)	0.406	0.03 (0.03-0.03)	0.432
PC_FA(18:0)	13.31 (11.70-15.42)	14.74 (10.03-15.51)	1.000	14.17 (11.55-16.59)	0.858
PC_FA(18:1)	13.48 (10.66-14.85)	15.40 (12.63-17.11)	0.138	15.51 (14.87-16.12)	0.078
PC_FA(18:2)	37.23 (37.09-38.43)	35.50 (34.80-36.60)	0.128	35.85 (33.13-36.77)	0.065
PC_FA(18:3)	0.64 (0.25-0.83)	0.65 (0.48-1.07)	0.929	0.03 (0.03-0.33)	0.043
PC_FA(20:2)	0.02 (0.02-0.02)	0.02 (0.02-0.02)	1.000	0.02 (0.02-0.02)	1.000
PC_FA(20:3)	0.02 (0.02-0.02)	0.02 (0.02-0.21)	0.297	0.02 (0.02-0.02)	0.640
PC_FA(20:4)	2.52 (1.65-3.02)	2.19 (1.31-2.88)	0.927	1.38 (0.10-2.72)	0.060
PE_FA(16:0)	9.23 (8.38-9.26)	9.31 (8.52-10.50)	0.615	9.86 (8.89-10.10)	0.438
PE_FA(16:1)	0.02 (0.02-0.21)	0.02 (0.02-0.38)	0.971	0.02 (0.02-0.02)	0.429

PE_FA(17:0)	0.03 (0.03-0.03)	0.03 (0.03-0.59)	0.145	0.03 (0.03-0.03)	1.000
PE_FA(18:0)	31.85 (31.60-33.03)	31.42 (30.45-32.54)	0.493	32.64 (31.86-33.81)	0.944
PE_FA(18:1)	13.56 (12.49-13.80)	15.96 (12.93-17.50)	0.063	15.41 (14.94-16.73)	0.012
PE_FA(18:2)	35.55 (34.17-36.30)	33.06 (32.78-37.23)	0.588	33.82 (32.70-38.54)	0.958
PE_FA(18:3)	0.05 (0.05-0.05)	0.05 (0.05-0.57)	0.296	0.05 (0.05-0.05)	0.758
PE_FA(20:1)	0.03 (0.03-0.03)	0.03 (0.03-0.03)	1.000	0.03 (0.03-0.03)	1.000
PE_FA(20:2)	0.02 (0.02-0.02)	0.02 (0.02-0.02)	1.000	0.02 (0.02-0.02)	1.000
PE_FA(20:3)	0.04 (0.04-0.04)	0.04 (0.04-0.04)	0.406	0.04 (0.04-0.04)	0.432
PE_FA(20:4)	9.30 (8.42-10.72)	8.32 (7.86-9.50)	0.910	7.77 (4.83-8.34)	0.106
PE_FA(22:4)	0.04 (0.04-1.08)	0.50 (0.04-0.92)	0.925	0.04 (0.04-0.41)	0.706
PE_FA(22:5)	0.02 (0.02-0.02)	0.02 (0.02-0.02)	1.000	0.02 (0.02-0.02)	0.322
SM_FA(14:0)	0.09 (0.08-0.10)	0.08 (0.06-0.09)	0.987	0.01 (0.01-0.05)	0.034
SM_FA(16:0)	5.11 (3.67-5.49)	3.60 (3.48-4.28)	0.121	4.74 (4.33-5.77)	0.876
SM_FA(18:0)	1.00 (0.74-1.10)	0.66 (0.55-0.70)	0.130	0.97 (0.78-1.35)	0.550
SM_FA(18:1)	0.39 (0.33-0.48)	0.28 (0.26-0.34)	0.154	0.53 (0.35-0.57)	0.348
SM_FA(20:0)	30.21 (26.29-31.33)	29.21 (24.63-30.95)	0.829	26.41 (23.08-29.28)	0.283
SM_FA(20:1)	0.97 (0.86-1.22)	1.05 (0.89-1.07)	0.999	0.73 (0.72-0.76)	0.007
SM_FA(22:0)	43.77 (39.41-48.54)	46.54 (44.58-47.68)	0.318	43.54 (42.60-46.25)	0.940
SM_FA(22:1)	6.25 (5.91-7.27)	6.89 (6.34-7.96)	0.154	5.59 (5.07-5.75)	0.158
SM_FA(24:0)	11.92 (8.35-12.67)	9.19 (7.11-11.66)	0.651	14.21 (9.86-17.65)	0.592
SM_FA(24:1)	1.94 (1.59-1.96)	2.20 (1.90-2.34)	0.198	1.76 (1.60-2.55)	0.774
SM_FA(26:0)	0.00 (0.00-0.00)	0.00 (0.00-0.00)	1.000	0.00 (0.00-0.00)	1.000
SM_FA(26:1)	0.01 (0.01-0.07)	0.05 (0.01-0.07)	0.841	0.03 (0.01-0.07)	0.939
TAG_FA(12:0)	0.16 (0.13-0.18)	0.17 (0.15-0.18)	0.795	0.10 (0.10-0.13)	0.015
TAG_FA(14:0)	3.89 (3.38-4.27)	4.24 (3.84-4.99)	0.365	2.01 (1.75-2.67)	0.003
TAG_FA(14:1)	0.20 (0.17-0.28)	0.32 (0.16-0.39)	0.559	0.11 (0.09-0.19)	0.041
TAG_FA(15:0)	0.83 (0.77-1.04)	1.07 (0.74-1.28)	0.239	0.63 (0.57-0.71)	0.022
TAG_FA(16:0)	29.89 (27.52-33.40)	30.02 (28.11-31.37)	0.861	36.41 (35.46-38.20)	<0.001
TAG_FA(16:1)	3.74 (3.20-4.90)	3.89 (3.55-4.77)	0.918	2.86 (2.71-3.46)	0.047
TAG_FA(17:0)	0.82 (0.73-0.95)	1.03 (0.85-1.16)	0.011	0.58 (0.55-0.59)	<0.001
TAG_FA(18:0)	3.72 (3.39-3.81)	4.03 (3.82-4.21)	0.120	4.03 (3.71-4.19)	0.417
TAG_FA(18:1)	27.71 (25.17-29.93)	30.67 (28.13-34.82)	0.032	35.05 (32.44-35.92)	<0.001
TAG_FA(18:2)	23.19 (20.60-25.16)	17.13 (16.40-20.20)	0.001	14.91 (13.54-16.16)	<0.001

TAG_FA(18:3)	3.61 (3.20-4.26)	3.63 (3.56-4.22)	0.572	1.97 (1.83-2.01)	<0.001
TAG_FA(20:0)	0.12 (0.11-0.12)	0.13 (0.11-0.13)	0.765	0.14 (0.12-0.16)	0.945
TAG_FA(20:1)	0.36 (0.27-0.42)	0.62 (0.48-0.77)	0.015	0.30 (0.22-0.44)	0.833
TAG_FA(20:2)	0.30 (0.27-0.34)	0.37 (0.32-0.47)	0.105	0.19 (0.16-0.26)	0.021
TAG_FA(20:3)	0.19 (0.17-0.22)	0.27 (0.25-0.31)	0.003	0.16 (0.11-0.18)	0.145
TAG_FA(20:4)	0.22 (0.19-0.29)	0.17 (0.13-0.21)	0.100	0.20 (0.13-0.25)	0.298
TAG_FA(20:5)	0.03 (0.02-0.03)	0.03 (0.02-0.03)	0.563	0.02 (0.02-0.04)	0.557
TAG_FA(22:1)	0.02 (0.02-0.02)	0.02 (0.02-0.02)	0.833	0.02 (0.01-0.02)	0.012
TAG_FA(22:4)	0.09 (0.08-0.10)	0.08 (0.07-0.09)	0.419	0.07 (0.05-0.09)	0.098
TAG_FA(22:5)	0.12 (0.11-0.14)	0.11 (0.10-0.14)	0.519	0.12 (0.09-0.15)	0.657
TAG_FA(22:6)	0.05 (0.04-0.05)	0.04 (0.04-0.05)	0.460	0.04 (0.03-0.05)	0.802
Ratios					
PA:OA	1.20 (1.07-1.49)	1.10 (0.96-1.21)	0.148	1.12 (1.09-1.23)	0.429
SFA:PUFA	1.49 (1.39-1.52)	1.77 (1.71-1.89)	<0.001	2.16 (2.02-2.27)	<0.001
PA:PUFA	0.93 (0.91-1.01)	1.14 (1.02-1.18)	0.012	1.44 (1.25-1.51)	<0.001
OA:PUFA	0.79 (0.62-0.84)	1.11 (0.84-1.17)	0.014	1.25 (1.14-1.45)	<0.001
CE_PA:OA	0.43 (0.29-0.52)	0.35 (0.31-0.50)	0.914	0.36 (0.35-0.42)	0.780
CE_SFA:PUFA	0.53 (0.47-0.59)	0.61 (0.57-0.67)	0.200	0.83 (0.78-0.89)	<0.001
CE_PA:PUFA	0.34 (0.33-0.35)	0.38 (0.34-0.42)	0.312	0.56 (0.50-0.60)	<0.001
CE_OA:PUFA	0.79 (0.60-1.19)	1.11 (0.69-1.16)	0.640	1.65 (1.52-1.76)	0.016
DAG_PA:OA	0.71 (0.50-0.76)	0.57 (0.46-0.63)	0.124	0.69 (0.64-0.79)	0.827
DAG_SFA:PUFA	1.13 (1.10-1.31)	1.19 (1.07-1.44)	0.670	1.72 (1.71-1.81)	<0.001
DAG_SFA18C:PUFA18C	0.12 (0.11-0.14)	0.15 (0.15-0.16)	0.057	0.21 (0.17-0.21)	<0.001
DAG_PA:PUFA	0.85 (0.75-0.86)	0.78 (0.72-0.99)	0.741	1.34 (1.29-1.41)	<0.001
DAG_PA:PUFA18C	0.86 (0.78-0.89)	0.82 (0.76-1.03)	0.602	1.39 (1.35-1.52)	<0.001
DAG_OA:PUFA	1.13 (1.10-1.32)	1.58 (1.36-1.82)	0.032	2.07 (1.68-2.29)	<0.001
FFA_PA:OA	2.56 (1.80-3.77)	2.17 (1.50-2.35)	0.222	1.59 (1.46-1.72)	0.018
FFA_SFA:PUFA	3.61 (3.01-4.43)	3.99 (3.85-4.76)	0.328	4.84 (4.35-5.38)	0.120
FFA_PA:PUFA	2.02 (1.67-2.56)	2.31 (2.25-2.66)	0.196	2.97 (2.66-3.31)	0.012
FFA_OA:PUFA	0.84 (0.68-1.06)	1.26 (1.14-1.55)	0.001	1.87 (1.82-1.93)	<0.001
LPC_PA:OA	1.72 (1.29-1.92)	1.31 (1.25-1.62)	0.274	1.24 (1.14-1.43)	0.037
LPC_SFA:PUFA	1.15 (0.99-1.26)	1.33 (1.11-1.39)	0.483	1.14 (1.08-1.26)	0.976
LPC_PA:PUFA	0.70 (0.66-0.83)	0.84 (0.74-0.88)	0.401	0.74 (0.70-0.82)	0.878

LPC_OA:PUFA	0.48 (0.39-0.51)	0.59 (0.56-0.66)	0.021	0.60 (0.57-0.62)	0.009
LPE_PA:OA	1.05 (0.85-1.35)	0.92 (0.73-0.98)	0.236	0.64 (0.59-0.83)	0.076
LPE_SFA:PUFA	5.47 (4.23-6.35)	6.00 (3.92-7.58)	0.791	3.79 (3.55-4.53)	0.801
LPE_PA:PUFA	1.89 (1.26-2.21)	2.07 (1.40-2.35)	0.852	1.46 (1.30-1.56)	0.886
LPE_OA:PUFA	1.80 (1.40-2.10)	2.18 (1.89-2.68)	0.986	2.19 (2.02-2.69)	0.822
PC_PA:OA	2.47 (2.14-2.91)	1.99 (1.88-2.60)	0.188	2.19 (2.04-2.34)	0.218
PC_SFA:PUFA	1.13 (1.09-1.19)	1.21 (1.10-1.27)	0.503	1.26 (1.18-1.34)	0.031
PC_PA:PUFA	0.82 (0.71-0.90)	0.80 (0.73-1.01)	0.826	0.90 (0.80-1.02)	0.291
PC_OA:PUFA	0.33 (0.26-0.38)	0.39 (0.33-0.46)	0.142	0.42 (0.39-0.42)	0.043
PE_PA:OA	0.65 (0.62-0.74)	0.60 (0.51-0.76)	0.393	0.60 (0.56-0.64)	0.202
PE_SFA:PUFA	0.91 (0.88-0.92)	0.95 (0.92-0.98)	0.054	1.02 (0.98-1.04)	<0.001
PE_PA:PUFA	0.20 (0.18-0.20)	0.23 (0.19-0.24)	0.240	0.23 (0.21-0.25)	0.056
PE_OA:PUFA	0.30 (0.27-0.30)	0.36 (0.29-0.42)	0.042	0.36 (0.35-0.41)	0.004
TAG_PA:OA	1.08 (0.99-1.34)	1.02 (0.81-1.09)	0.150	1.04 (0.99-1.18)	0.831
TAG_SFA:PUFA	1.43 (1.31-1.56)	1.76 (1.65-2.01)	0.003	2.49 (2.37-2.62)	<0.001
TAG_SFA18C:PUFA18C	0.14 (0.13-0.16)	0.19 (0.18-0.21)	0.003	0.24 (0.23-0.26)	<0.001
TAG_PA:PUFA	1.05 (1.03-1.22)	1.29 (1.20-1.48)	0.014	2.03 (1.96-2.15)	<0.001
TAG_PA:PUFA18C	1.10 (1.06-1.33)	1.35 (1.26-1.56)	0.022	2.13 (2.10-2.23)	<0.001
TAG_OA:PUFA	0.97 (0.90-1.14)	1.42 (1.10-1.62)	0.006	1.98 (1.72-2.22)	<0.001

Results are presented as median (P25-P75). CE : cholesteryl esters. CER : ceramides. DAG : diacylglycerols. DCER : dihydroceramides. FFA : free fatty acids. HCER : hesoxylceramides. LCER : lactosylceramides. LPC : Lysophosphatidylcholines. LPE : lysophosphatidylethanolamines. PC : phosphatidylcholines. PE : phosphatidylethanolamines. SM : sphingomyelins. TAG : triacylglycerols. PA : palmitic acid. OA : oleic acid. SFA : saturated fatty acid. MUFA : monounsaturated fatty acid. PUFA : polyunsaturated fatty acid. 18C : 18C-long chain fatty acid.
